# Data Driven Classification Using fMRI Network Measures: Application to Schizophrenia

**DOI:** 10.3389/fninf.2018.00071

**Published:** 2018-10-30

**Authors:** Pantea Moghimi, Kelvin O. Lim, Theoden I. Netoff

**Affiliations:** ^1^Department of Biomedical Engineering, University of Minnesota, Minneapolis, MN, United States; ^2^Department of Psychiatry, University of Minnesota, Minneapolis, MN, United States

**Keywords:** resting-state fMRI, classification, network measures, double cross validation, prewhitening, schizophrenia

## Abstract

Using classification to identify biomarkers for various brain disorders has become a common practice among the functional MR imaging community. Typical classification pipeline includes taking the time series, extracting features from them, and using them to classify a set of patients and healthy controls. The most informative features are then presented as novel biomarkers. In this paper, we compared the results of single and double cross validation schemes on a cohort of 170 subjects with schizophrenia and healthy control subjects. We used graph theoretic measures as our features, comparing the use of functional and anatomical atlases to define nodes and the effect of prewhitening to remove autocorrelation trends. We found that double cross validation resulted in a 20% decrease in classification performance compared to single cross validation. The anatomical atlas resulted in higher classification results. Prewhitening resulted in a 10% boost in classification performance. Overall, a classification performance of 80% was obtained with a double-cross validation scheme using prewhitened time series and an anatomical brain atlas. However, reproducibility of classification within subjects across scans was surprisingly low and comparable to across subject classification rates, indicating that subject state during the short scan significantly influences the estimated features and classification performance.

## Introduction

Schizophrenia is a debilitating disease that affects between 0.25 and 0.64% of the adult US population according to (The National Institute of Mental Health, 2018). One hypothesis about the cause of schizophrenia is the “disconnectivity hypothesis” (Friston, 1998) which posits that the normal pattern of connectivity between distinct regions of the brain is affected. This hypothesis has been studied extensively over the past decade using Functional Magnetic Resonance Imaging (fMRI). fMRI provides a unique means to study schizophrenia because it is non-invasive and unlike Electroencephalography (EEG) can image deep brain structures, such as the thalamus. A reliable biomarker of the disease could help clinicians with diagnosis, measure efficacy of a therapy, and identify prodromal state. Biomarkers may also provide insight into the mechanism of the disease and could guide researchers to developing novel therapeutic interventions.

Finding biomarkers that are replicable across different studies and patient populations has been challenging (Pettersson-Yeo et al., 2011). Different studies have found abnormal patterns of connectivity in different regions of the brain. For example, the thalamus is often implicated, but some studies have reported hyperconnectivity between thalamus and other brain areas (Wolf et al., 2009; Skudlarski et al., 2010; Zhang et al., 2012; Atluri et al., 2015) while others have found hypoconnectivity (Andreasen et al., 1996; Zhou et al., 2007; Tu et al., 2010).

There are several approaches to generating features from fMRI to identify biomarkers: (1) a univariate approach, which quantifies characteristics of each brain region separately and compares that across the patient and healthy groups (Shi et al., 2007; Bassett et al., 2012), (2) a bivariate approach, which quantifies interaction between each pair of regions in the brain and searches for region pairs that are discriminating across patient and healthy groups (Lynall et al., 2010; Bassett et al., 2012; Tang et al., 2012; Su et al., 2013; Guo et al., 2013; Arbabshirani et al., 2014a; Kim et al., 2016), and (3) a multivariate approach, which quantifies more complex interactions between all regions, typically using graph theoretic measures (Lynall et al., 2010; van den Heuvel et al., 2010, 2013; Bassett et al., 2012; Anderson and Cohen, 2013; Fekete et al., 2013; Singh and Bagler, 2016). The univariate approach, while simpler and less computationally demanding, does not capture anomalies in interactions between different regions. The bivariate approach captures pairwise interactions between different brain regions but is not capable of mining more complex structures resulting from those pairwise interactions. Furthermore, the bivariate approach results in a high dimensional feature space to choose from, making the problem of finding robust biomarkers computationally more challenging. The multivariate approach while capturing regional interactions, reduces the feature set to a set of measures that capture complex patterns of interactions between regions without the need to analyze each pairwise interaction separately. Moreover, recent studies have demonstrated that the multivariate approaches have more discriminating power (Venkataraman et al., 2010; Atluri et al., 2013).

The discriminating power of a classifier can be quantified either by using statistical tests (e.g., Lynall et al., 2010; van den Heuvel et al., 2010, 2013) or by classification accuracy (e.g., Castro et al., 2011; Bassett et al., 2012; Anderson and Cohen, 2013; Arbabshirani et al., 2013; Singh and Bagler, 2016). Statistical tests measure the distance of means between the two groups. If it is a parametric test, this distance is measured in the standard error of the means of the two groups. Statistical significance depends on the number of subjects, as increasing the number of subjects can increase the power to detect subtle differences in their means. On the other hand, classification performance measures the accuracy of the classifier. Significant statistical difference between two groups can be measured but may still have nearly random classification performance. Moreover, statistical tests rely on the difference between means of the two groups, and are therefore sensitive to small changes in the means caused by outlier data points. Classifiers such as support vector machines (SVM) on the other hand are more robust to outliers (Vapnik, 1995; Bishop, 2006). Therefore, classification accuracy is a better measure of the value of a classifier in a clinical setting. Furthermore, classifiers provide a model that can be directly used to make predictions about new datasets.

The divergent, and sometimes inconsistent, biomarkers proposed for schizophrenia can be attributed to multiple factors. First, typical sample size are low (average 38 subjects based on sample sizes reported in Pettersson-Yeo et al., 2011). Second, different studies use different pre-processing steps. Third, tests that identify biomarkers that are statistically significantly different vs. those that use classification performance put different values on the performance of the features. Fourth, biomarker validation is different across studies and some findings may be more robust and generalize better than others. Finally, there is the issue of heterogeneity of the studied disorder.

In this study we examined predictive power of several multivariate biomarkers, but also explored how such predictive power can be affected by pre-processing steps and the biomarker discovery method. This work extends previous work in four important ways. First, we used a large sample (170 subjects). Second, we used a purely data driven approach to identifying biomarkers. We used a comprehensive set of multivariate graph theoretic measures and used data driven algorithms to find which ones have more discriminating power. Third, we used an alternative pre-processing pipeline where functional activity was prewhitened to remove spurious cross correlation between voxels. Moreover, we compared predictive power of two methods of defining brain regions: extracting definition of brain regions based on functional activity of the subjects themselves, compared to using a conventional anatomical atlas. And fourth, we used a rigorous double cross-validation method to discover biomarkers and report their prediction accuracies to improve generalization across different patient populations.

In this study 6 min of resting state fMRI data from subjects with schizophrenia and healthy control subjects were analyzed. All time series were first prewhitened, then used to construct a functional atlas. Regions of the functional atlas as well as an anatomical atlas commonly known as the AAL atlas (Tzourio-Mazoyer et al., 2002) were used to construct unweighted graphs for each subject, and several multivariate graph theoretic measures were calculated. The measures were then used to classify patients from healthy controls using SVMs. Most informative features were identified and used to report classification performance in a double cross-validation scheme where separate sets of subjects were used for feature selection and classification respectively.

## Materials and Methods

### Participants

A total of 170 subjects participated in this study: 52 chronic (17 female, age: *M* = 37.0, *SD* = 10.8) and 30 first episode (8 female, age: *M* = 25.7, *SD* = 7.1) subjects with schizophrenia. Since the patient group had different age distribution, two groups of healthy control subjects were recruited, where each healthy control group matched demographics of one of the patient groups: 55 control subjects (18 female, age: *M* = 38.0, *SD* = 11.9) to match the chronic group and 33 control subjects (9 female, age: *M* = 25.5, *SD* = 6.9) to match the first episode group (see Table 1 for detailed information on participants). All participants gave informed consent and were compensated for their participation. Schizophrenia patients were assessed for negative and positive symptoms using the Scale for Assessment of Negative Symptoms (SANS) and Scale for Assessment of Positive Symptoms (SAPS) (Andreasen and Olsen, 1982). All procedures were done in accordance with a University of Minnesota IRB approved protocol.

**Table 1:** Characteristics of study participants.

	Age	SANS	SAPS	Medication
Chronic Schizophrenia Patients (*N* = 52)	37.0(10.8)	33.0(14.3)	23.5(17.3)	1 Typical 38 Atypical 5 Both 4 No meds 4 N/A
Chronic Healthy Controls (*N* = 55)	38.0(11.9)	N/A	N/A	N/A
First Episode Schizophrenia Patients (*N* = 30)	25.7(7.1)	30.1(17.4)	25.3(16.9)	0 Typical 21 Atypical 0 Both 3 No meds 6 N/A
First Episode Healthy Controls (*N* = 33)	25.5(6.9)	N/A	N/A	N/A


*Mean (and SD) Demographic and Diagnostic Characteristics of participants. SANS, Scale for Assessment of Negative Symptoms; SAPS, Scale for Assessment of Positive Symptoms.*

For classification, chronic and first episode schizophrenic patients were collectively labeled as the schizophrenic group. Similarly, the two healthy control subjects populations were grouped into a single population labeled “healthy.”

### Imaging Data Acquisition and Pre-processing

Resting state fMRI was collected for 6 min from each participant as detailed in (Camchong et al., 2011; Atluri et al., 2015). Participants were instructed to remain as still as possible, stay awake and keep their eyes closed. Images were acquired using a Siemens Trio 3T scanner (Erlangen, Germany). Sequence parameters used in this study are as follows: gradient-echo echo-planar imaging (EPI) 180 volumes, repetition time (TR) 2 s, echo time (TE) 30 ms, flip angle 90°, 34 contiguous AC-PC aligned axial slices, voxel size 3.4 × 3.4 × 4.0 mm, matrix 64 × 64 × 34 totalling 139,264 voxels.

Participants were asked at the end of the scan whether or not they stayed awake during the scan. For the one patient that fell asleep during the scan the scan was repeated under awake conditions. Also, a T1-weighted anatomical image was acquired using a magnetization prepared rapid gradient-echo sequence. In addition, a field map was acquired and used to correct for geometric distortions introduced by field inhomogeneities: TR = 300 ms, TE = 1.91 ms/4.37 ms, flip angle = 55°, voxels size = 3.4 × 3.4 × 4.0 mm.

To remove recording artifacts and noise, register the data, and downsample to a manageable size, the raw fMRI data was preprocessed using FEAT and MELODIC from the FSL software package as follows. First, the first three volumes were excluded from each subject scan to account for magnetization stabilization. The subsequent scans were then motion corrected, B0 field map unwarped, and corrected for slice scan time. Non-brain portions of the images were removed and a spatial smoothing kernel was applied to the dataset (6 mm full-width half-maximum). The images were then grand mean and intensity normalized and temporally filtered between 0.01 and 0.08 Hz. All images were then registered to the MNI152 space. To remove noise introduced by head motion, respiration, cardiac pulsation, and scanner artifacts, probabilistic independent component analysis (PICA) (Beckman and Smith, 2004) was used. Spatial and temporal characteristics of noise components are described in MELODIC manual^1^ and previous methodological reports (Kelly et al., 2010; Xu et al., 2014). The dataset was then resampled to 3 × 3 × 3 mm, resulting in 47640 voxels per volume. Time series for each voxel consisted of 177 time points, separated by 2 s, lasting 5.9 min in duration.

### Functional Parcellation

Functional parcellation is the process of grouping voxels with similar functional activity together to form regions using data-driven algorithms. Functional parcellation uses cross-correlation between the voxel time series to identify voxels with similar functional activity. Cross-correlation metrics are influenced by both the correlation between the two data sets as well as the autocorrelation within each data set. Therefore, to isolate the interactions with the cross-correlation, it should be applied to time series that are stationary and have no autocorrelation, characteristics of white noise. However, BOLD time series are typically non-stationary and are highly autocorrelated leading to spuriously high cross-correlations (Christova et al., 2011). For accurate functional maps it is important to remove these factors that lead to spuriously high correlation values. One approach to removing non-stationary and autocorrelated trends from the time series is “prewhitening” (Christova et al., 2011). This approach has previously been used to find biomarkers for Post Traumatic Stress Disorder (PTSD) and shown to enhance classification performance of the biomarkers (Christova et al., 2015). Therefore, we prewhitened all time series prior to constructing the functional atlas. To prewhiten the time series from voxel *i x_i_(t)*, the Fourier transform of the time series, *X_i_(f)*, was calculated and divided by the absolute value of the spectrum (Equation 1), so that similar to white noise, the amount of power in each frequency band was equal and the power spectrum became flat. Autocorrelation of the whitened signal is calculated as inverse Fourier transform of the resultant flat spectrum which is an impulse. Fourier transform of each time series was calculated as its 177 point Discrete Fourier transform (DFT) using MATLAB’s fft() function.

(1) $$\begin{array}{c} x_{i}(t)\leftarrow \to X_{i}\left(f\right)\\ {X^{W}}_{i}(f)=X_{i}(f)/|X_{i}\left(f)|\leftarrow \to {x^{W}}_{i}(t\right) \end{array}$$

The resultant spectrum, *X^W^_i_(f)*, was then transformed back into the time domain *x^W^_i_(t)* to make a prewhitened data set. While this approach uses an a-causal approach to prewhitening, unlike fitting an ARMA model, it is highly efficient and when only the zero time lag correlation is measured, this approach can be used for undirected similarity measures.

**FIGURE 1:**
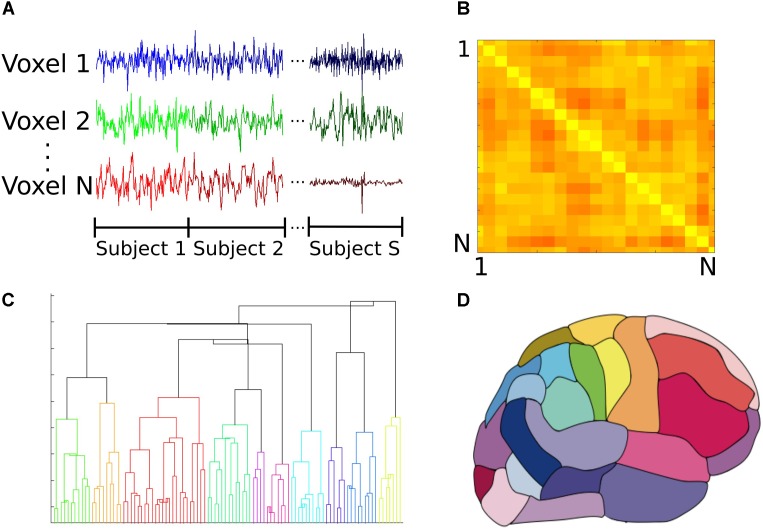
Construction of the group functional atlas. **(A)** In order to combine datasets from individual subjects, time series from all control subjects were concatenated for each voxel. Each individual dataset consisted of 47640 voxels and 177 time points. **(B)** A toy example illustrating how the adjacency matrix is constructed from pairwise cross correlations between the time series of all pairs of *N* voxels. The *i*th row and *j*th column equals cross correlation between the *i*th and *j*th voxels. Lighter and darker colors correspond to higher correlation and lower correlation values respectively. For our dataset, this was a 47640 × 47640 symmetric matrix which was used to calculate pairwise correlation distance between all voxels. **(C)** Dendrogram illustrating cluster distances. A dendrogram contains all the information about membership of each datapoint at each stage of hierarchical clustering. At the bottom of the dendrogram, each single data point constitutes a single cluster. At each stage of the hierarchy, the pair of clusters that are most similar as evaluated by the linkage criterion are merged to form bigger clusters. Eventually, at the top of the hierarchy all data points are merged to form a single cluster. **(D)** Segmentation based on dendrogram. After cutting the dendrogram at the selected scale, i.e., the desired number of clusters or regions, a parcellation of the brain is produced based on which voxels belongs to the same cluster. This parcellation is used as the functional atlas for the rest of our analyses.

The functional atlas was constructed at group level by combining scans from the control subjects. To combine individual scans, we concatenated time series from all the subjects, to obtain a single time series per voxel (Figure 1A). We then calculated the correlation adjacency matrix between the voxels (Equation 2), where *X_W_* is the time series matrix, with each column corresponding to one time series and each row corresponding to the BOLD signals across voxels at a single time point. For a dataset consisting of N voxels, the correlation adjacency matrix is an *N × N* symmetric matrix where value of the *i^th^* row and *j^th^* column is the Pearson correlation coefficient (Altman, 2006) between time series of voxels *i* and *j* (Figure 1B). Pairwise correlation values were then used to calculate pairwise correlation distance between voxels, which is equal to 1 minus the correlation coefficient between the pair, and ranges from 0 to 2.

(2) $$\begin{array}{c} \Sigma ={X^{T}}_{W}.X_{W}\\ C={\left(diag\left(\Sigma \right)\right)}^{-1/2}.\Sigma .{\left(diag\left(\Sigma \right)\right)}^{-1/2} \end{array}$$

To construct the atlas, we used the agglomerative hierarchical clustering algorithm, with Ward’s minimum variance method as the linkage criterion (Ward, 1963; Tan et al., 2006). In this algorithm, at first each voxel is treated as a single region or cluster. Then, the pair of clusters with minimum within cluster distance among all the pairs are grouped together to form bigger clusters. This process is repeated until all the voxels are merged into a single cluster. A dendrogram can be generated that shows arrangement of the clusters at each stage of hierarchical clustering (Figure 1C). The final cluster assignments for each data point is then obtained by ‘cutting’ the dendrogram at a desired scale, for example 90 regions (Figure 1D). To obtain contiguous regions, a spatial constraint was enforced when constructing the dendrogram that allowed two clusters to be merged only if they contained spatially neighboring voxels, and therefore their merger would result in a contiguous region. Our choice of the Ward’s linkage method was based on an exploratory analysis of different parcellation methods described in detail elsewhere (Moghimi et al., 2017).

To compare classification accuracy with an anatomical atlas, we constructed a functional group atlas with 90 regions to compare to the commonly used Automated Anatomical Labeling (AAL) atlas, which also has 90 regions excluding cerebellum (Tzourio-Mazoyer et al., 2002). Number of regions for the functional atlas was chosen to be similar to that of the AAL to make a direct comparison between the two atlases. Distribution of size of regions was also similar across the two atlases (Moghimi et al., 2017). Borders between the two regions do not align, however, and regions of the functional atlas are not anatomically meaningful since they were constructed using functional activity. The location of each region in the functional atlas can be reported in terms of the AAL regions it overlaps with.

### Network Model

**FIGURE 2:**
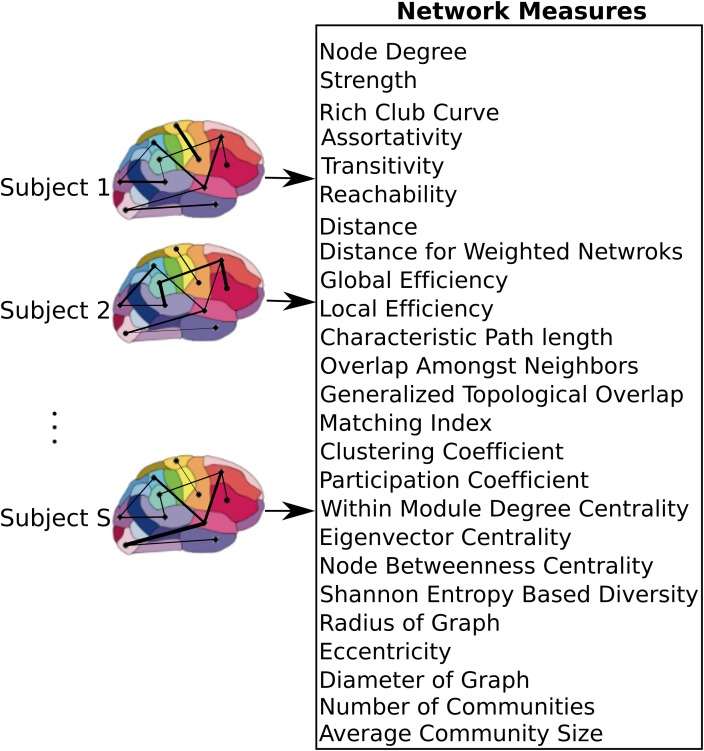
Network level model of the brain. After applying the atlas to each individual dataset, time series of voxels within each region were averaged resulting in a single time series per region. These regions were used as nodes of the graph, where the link between each pair of nodes was weighted by the correlation coefficient between the nodes. A set of measures that capture network characteristics were then calculated for each subject.

After constructing the functional atlas, a graph model of the brain was constructed for each subject by first applying the atlas to the individual datasets. Time series of all voxels within a single region were averaged to obtain a single time series per region. Pairwise Pearson correlation coefficient between the regions was then calculated and used to construct a weighted undirected graph, where each region constituted one node and the links were weighted by the correlation coefficient value between nodes (Figure 2). Calculation of the network measures requires all the weights to be non-negative, so negative weights were set to zero. There is currently no general consensus over the cause of negative correlation coefficients (Chen et al., 2011) and we observed that only 2 ± 3% of all cross correlations were negative. Several measures are specific to binary graphs (Supplementary Table 1). In order to construct a binary graph, weights that were below a threshold were set to zero and weights above the threshold were set to one. The threshold was chosen to obtain a binary graph with 30% connection density (Lynall et al., 2010). In addition, some measures required the graph to be divided into communities (Supplementary Table 1) and the information about the community membership was required for their calculation. To divide the graph into communities, the Louvain method for community detection (Reichardt and Bornholdt, 2006; Ronhovde and Nussinov, 2009) was used. After constructing the weighted and binary graphs, several graph theoretic measures (Bullmore and Sporns, 2009; Rubinov and Sporns, 2010b) were calculated (see Figure 2 and Supplementary Table 1 for a list of the measures), using the Brain Connectivity Toolbox (Rubinov and Sporns, 2010a,b). Some measures that required specification of extra parameters, as summarized in Supplementary Table 1.

Graph theoretic measures can capture characteristics of each node (producing one value per node), each pair of nodes (producing one value per node-pair), or the entire network as a whole (producing one value per network). In addition, for each measure, its average and standard deviation across all regions were also used as separate measures. As a result, each node measure produced 90 + 2 features (one feature per node plus mean and standard deviation across all nodes). Similarly, measures that captured characteristics of node pairs each produced 4005 + 2 features since there are 4005 node pairs in a graph with 90 nodes. Global network measures each produced a single feature. If for any particular measure, the values for some nodes or pairs of nodes were not calculable due to structure of the graph, those values were excluded from the feature set. With 10 node measures, 6 node-pair measures, and 9 global measures, we computed a total of 19000+ network measures.

### Classification

**FIGURE 3:**
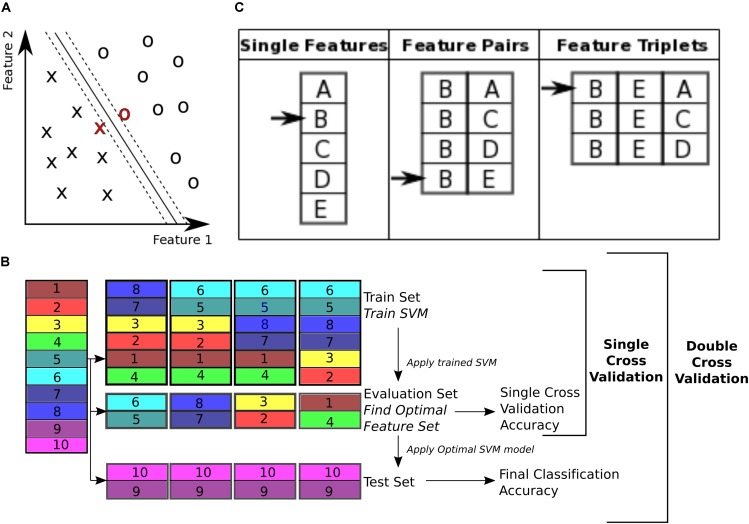
Classification. **(A)** Support vector machine is a supervised learning classifier that maximizes the margin between the separating hyperplane (continuous black line) and data points. Data points closest to the hyperplane are the support vectors. In this toy example, the data consists of two features. Our dataset consists of 170 data points, in a 19000+ dimensional space. The hyperplane is characterized by a set of weights (W) and constant (b) and projects the dataset onto a single dimension. **(B)** Double cross validation divides the subjects into three subsets. The *training* and *validation* subsets were used for optimizing the feature set, and the *test* subset is used for calculating classification performance. Different subsets are determined in a 5-fold cross validation division scheme, where the entire dataset is divided into 5 equal size subsets and each subset is used once as the *test* subset. In this toy example, subjects 9 and 10 are used as the *test* set, while the rest of the subjects are used for feature selection. For feature selection a 4-fold cross validation scheme is used, where the subjects are partitioned into 4 equal size groups and each group is used once as the *validation* subset. 4 SVM models are trained for each *train* subset, and performance of each set of features is averaged across the 4 classifiers. Features that perform better on average are then chosen to be tested on the *test* set. **(C)** Sequential forward selection (SFS) algorithm is demonstrated in a toy example. First, performance of every single feature is calculated by training a SVM using that feature only on the *train* subset and applying the weights on the *validation* subset. The single feature with the highest performance is picked (feature B in this example, left column). Subsequently, performance of combination of feature B with all the remaining features is calculated, by training an SVM using each feature pair separately on the *train* subset and applying the weights to the *validation* subset. Feature pair with the highest performance is then selected [features (B, E) in this example, middle column]. The selected feature pair is then combined with all the remaining features to form feature triplets, performance of each is then calculated through the same cross validation procedure. The feature triplet with the highest performance is then picked (features (B, E, A) in this example, right column).

To classify control subjects from schizophrenia subjects we used SVM (Vapnik, 1995; Bishop, 2006). SVMs are robust to presence of noisy data points because they maximize the classification margin (Figure 3A). There are two free parameters for an SVM that need to be set by the experimenter: box constraint (*C* value) and kernel. We used a *C* value equal to 1, and a linear kernel. We also tried *C* values equal to [0.1, 10] and radial basis function (RBF) (sigma = 1), quadratic, and polynomial (degree = 3) kernels.

The 19000+ graph theoretic measures were used as features for classification of 170 subjects into either control or schizophrenic. Using this feature set for classification of a data set poses two challenges for classification. The first challenge is that the feature set is orders of magnitude larger than the number of subjects (a problem called ‘the curse of dimensionality’) (Jain et al., 2000). This forces the classifier to pick up patterns that are specific to the subjects that are used for its training and therefore are not generalizable to other subjects, a phenomenon called ‘overfitting’ (Clarke et al., 2008). The second challenge is that not all features are equally informative to the classifier (Guyon and Elisseeff, 2003). We need to know which features are contributing more to the classification process in order to extract effective biomarkers. Therefore, we need to reduce dimensionality of the data by selecting an optimal or sub-optimal subset of features for classification. Here we used SVM for both feature selection and classification. To ensure the optimized feature set is generalizable across subjects, we used a double cross validation scheme (Filzmoser et al., 2009; Sundermann et al., 2014).

To perform double cross validation, the subject set was randomly partitioned into three separate subsets: *train*, *validation*, and *test* subsets (illustrated in Figure 3B). The *train* subset was used to train an SVM model. The model performance was then validated on the validation subset. The *training* and *validation* subsets were used to iteratively optimize feature selection and SVM parameters. This ensures that the final performance is not influenced by the optimization, and reflects performance of the features more robustly than a single cross validation scheme, which uses the optimal SVM model to classify the validation subset itself.

The subject set (170 subjects) was divided into five randomly chosen subsets of equal size (34 subject each) and used in a 5-fold cross validation (Efron and Gong, 1983; Efron, 1983). One fold was left out to be used as the *test* subset and the rest were used for the SVM model optimization. For the feature selection, the *training* and *validation* folds were shuffled 4 times and used iteratively to select features that performed best on the validation set (Figure 3B). This performance is reported as the single cross validation performance. Once the features were selected the SVM was trained using both the *train* and *validation* folds and then applied to the *test* fold to obtain the final classification accuracy, (*(TP + TN)/T*), where *TP* is number of true positives, i.e., patients classified correctly, *TN* is number of true negatives, i.e., controls classified correctly, and *T* is the total number of subjects. This approach is similar to the leave one out cross validation (LOOCV) scheme, except that instead of leaving out a single subject, we leave out a single fold. In addition to classification accuracy, sensitivity (*TP/P*) and specificity (*TN/N*) (Fawcett, 2006) were also reported (*P* is the total number of patients and *N* is the total number of controls).

With 10 random partitions (*N_perm_*), each subject is used in the *test* subset 10 times. We calculated the proportion of times each subject was misclassified (*M*), a measure we term the “misclassification rate,” *MR = M/ N_perm_*. Correlation between misclassification rate (*MR*) and severity of symptoms for the patient population was calculated.

In order to see if choice of classification algorithm affects the classification performance, we compared performance of SVM to SVM with Adaboost (short for Adaptive Boosting) (Freund and Schapire, 1997; Yoav and Schapire, 1999) with a linear SVM (*C* value equal to 1), and 10 weak classifiers were trained.

To calculate classification accuracy due to chance, we repeated the classification process 10 times with randomly shuffled labels.

All analyses were implemented in MATLAB 2016b.

### Feature Selection

With 19000+ features, only a fraction are informative for classification and the others dilute classification power by causing the classifier to overfit. Therefore, it is beneficial to choose the subset of features that are the most informative. These were determined using a data driven greedy search procedure, called sequential forward selection (SFS) (Guyon and Elisseeff, 2003) (Figure 3C). First, the classification accuracy of each single feature alone was measured using SVM, cross validating across the *train* and *validation* subsets. Only features with prediction accuracy above 60% were used for the subsequent stages of the optimization, resulting in 1618 features on average. Then, the feature with the top performance was progressively combined with other features, selecting the combinations with highest accuracies, until a set of 40 features were selected. This method of feature selection is computationally expensive but it is more robust than simply selecting 40 features that independently have the highest performance. Many of the top features alone may have redundant information. This algorithm accounts for the combinatory effect of features. Moreover, while a feature might have low classification performance on its own, in combination with other features it can improve performance (Guyon and Elisseeff, 2003). The SFS algorithm is not guaranteed to find the globally optimal set of features that would maximize classification accuracy, but it is guaranteed to find a local optimum (Liu and Motoda, 2007).

The random partitioning into the 5-folds was performed 10 times resulting in 50 optimized feature sets, of 40 features each, and 50 prediction accuracies. To determine if inclusion of any feature in the feature set occurs more often than expected by chance, we calculated the probability of each feature appearing *n* times out of 50. The probability of each feature appearing once in each selected set is equal to the probability of drawing 40 random samples from a set of *F* items without replacement, which can be calculated with the hypergeometric distribution *P_Select,S_ = h(1| F, S, P)* where *F* is the total number of features used (*F* = 1618) (see feature selection section), *S* is the number of samples (*S* = 40), and *P* is the number with the desired property (*P* = 1). Given the probability of sampling each feature at random, we can then calculate the number of times that feature is expected to appear with *N_fold_* independent draws using a binomial distribution *Pr(n) = B(N_fold_, P_Select,S_)*. Features that appeared more frequently than predicted by chance were further analyzed.

To see if our feature selection algorithm improves classification accuracy for our dataset, we compared the accuracy achieved by the SFS algorithm to that of the best 40 features (independent feature selection) and top 40 features selected using Fisher’s linear discriminant analysis, also known as the Linear Discriminant Analysis (LDA) (Fisher, 1936; Bishop, 2006). LDA transforms the data into a space where the linear separation between the two classes is maximized. Calculation of weights for the linear transformation involves matrix inversion, which is not possible if the within-class scatter matrix is singular, depending on structure of the dataset. Therefore, Moore-Penrose pseudo-inverse of the matrix was calculated (Campbell and Meyer, 2008).

## Results

**FIGURE 4:**
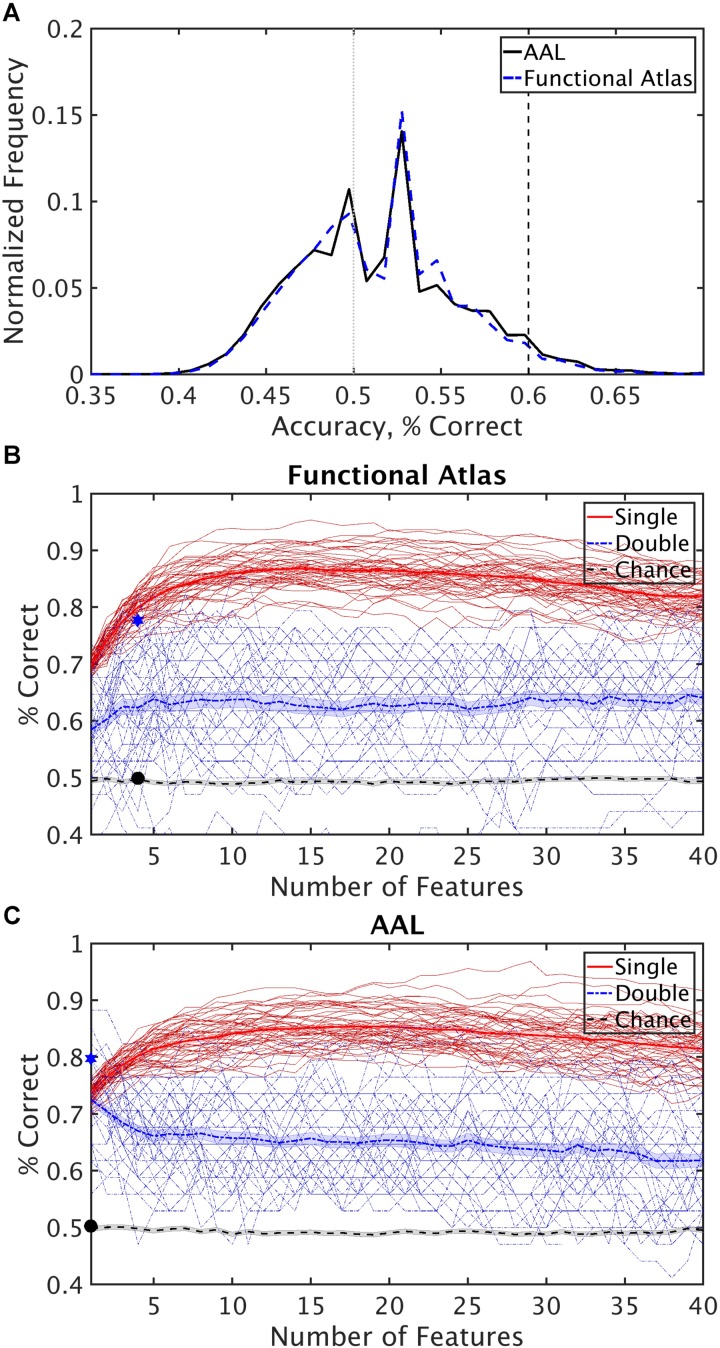
Classification performance. **(A)** Distribution of classification accuracy of single features, when used independently for classification, for the functional and AAL atlases. Dashed vertical lines mark the 60% accuracy threshold and 50% chance level. Features with less than 60% accuracy were not considered in the feature selection process. **(B)** Single and double cross validation classification performance as a function of number of features, using the functional atlas to define nodes of the network. The star marks classification accuracy when the most frequent four features were used to classify the entire subject set. The circle marks chance level performance for the same features. **(C)** Single and double cross validation classification performance as a function of number of features, using AAL atlas to define nodes of the network. The star marks classification accuracy when the most frequent single feature was used to classify the entire subject set. The circle marks chance level performance for the same feature.

Two atlases, the anatomical AAL atlas and a functional atlas constructed using time series from the control group, were used as region definitions for construction of brain networks. The functional activity in each region of the atlas was averaged and zero-lag cross correlation between the regions were used to construct undirected weighted graphs for each subject. Several graph theoretic measures were then calculated for each network and used as features for classification. This resulted in 19,000 features. To reduce the number of features, we selected features whose classification accuracy using linear SVM achieved was greater than 60% accuracy, reducing the total number of features used in optimization of combinations of features to, on average, 1618 features. A distribution of the single feature classification accuracy using the functional atlas and the AAL atlas is shown in Figure 4A.

The set of 1618 features is an order of magnitude larger than number of subjects (*N* = 170). In this high dimensional space, the classifier picks up on subtle variations that are specific to the subject set used for training the classifier, which generalizes poorly to unseen data. This phenomenon is called the curse of dimensionality (Jain et al., 2000). For robustness it is necessary to reduce dimensionality of the dataset before classification. To reduce dimensionality of the dataset, the top performing features with higher than 60% classification accuracy were combined through the (SFS) algorithm to find the combination that provided the best classification. Classification accuracy was calculated for single cross validation using features selected with the *training* and *validation* subsets. The final reported calculated classification accuracy was calculated by double cross validation where the optimized SVM model and features were then applied to a final *test* set not used in the feature selection optimization. Comparison of the single cross validation and the double cross validation performances for the functional atlas are shown in Figure 4B. The single cross validation results were consistent with previously reported classification rates with accuracy maximizing at 87% using 14 features, which is significantly above chance level (*p* < 0.001, two sample *t*-test). The double cross validation maximum accuracy was significantly lower, dropping to 64% peaking at 4 features, which is still significantly above chance (*p* < 0.001, two sample *t*-test), but about 20% lower than the single cross validation performance. However, the double cross validation rate is probably a more accurate estimate that would generalize to prospective studies. The high classification rate reported by the single cross validation can be accounted for by the overfitting using the feature selection optimization step. Results are also reported based on network analysis of brain areas using the AAL atlas in Figure 4C. The single cross validation performance maximized at 85% using 18 features, similar to the functional atlas. Double cross validation accuracy was 73% using only a single feature.

**FIGURE 5:**
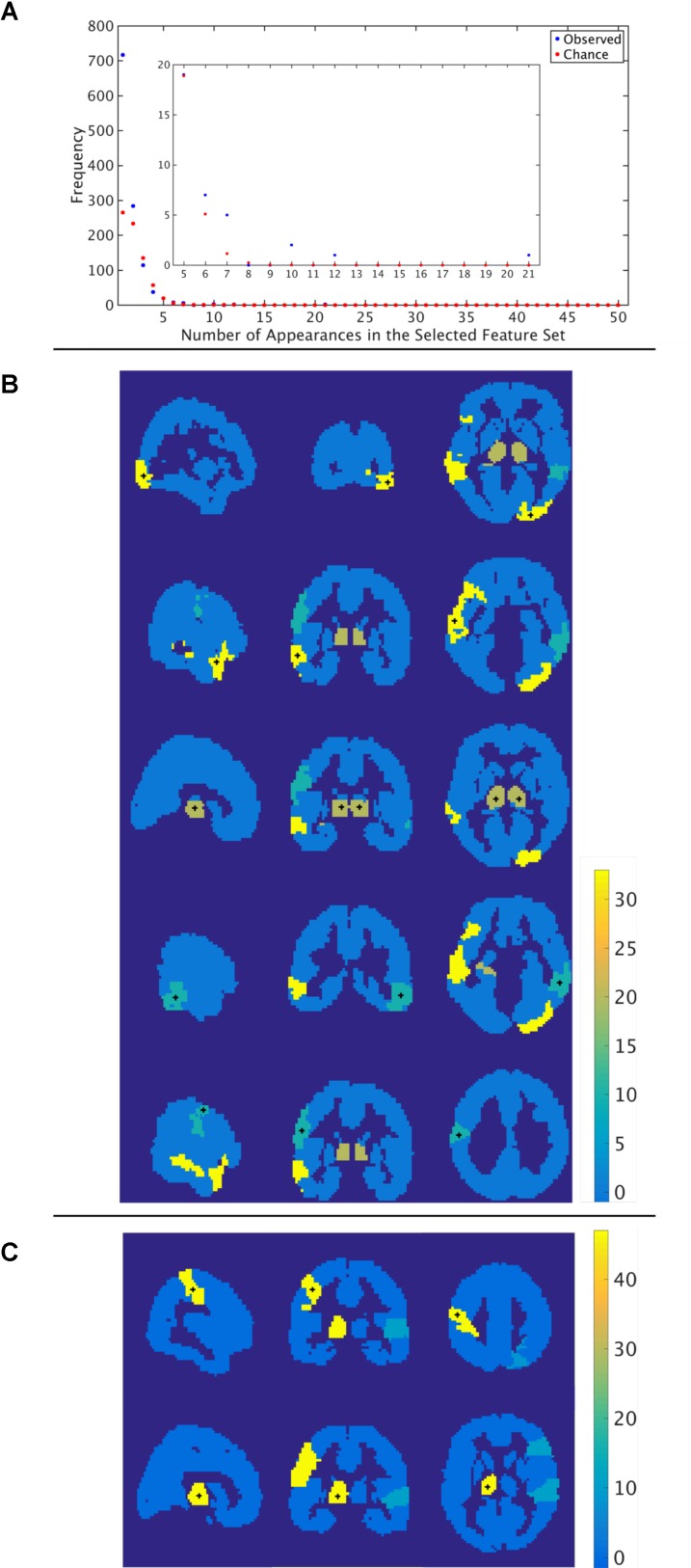
Most informative features. **(A)** Number of features (*y*-axis) vs. frequency of single features appearing in the 50 selected feature sets (*x*-axis) when features are calculated using the functional atlas. The inset is an expanded view of the most informative features. For example, one feature has appeared 21 times in the selected feature set. **(B)** Spatial maps showing where the most informative regions are for the functional atlas. The + marks center of the region. Colormap shows number of appearances of the region as one of the top 40 features. Left: Sagittal view, Middle: Coronal view, Left: Horizontal view. **(C)** Spatial maps showing where the most informative regions are for the AAL atlas. The + marks center of the region. Colormap shows number of appearances of the region as one of the top 40 features. Left: Sagittal view, Middle: Coronal view, Left: Horizontal view.

Through the feature selection process we identified the top 40 most informative features, which was repeated through 5-fold cross validation 50 times. Therefore, each feature could appear in the selected feature set from 0 to 50 times. To identify those features that were selected more often than would be expected if selected randomly, we calculated the probability of a feature being selected *n* times due to chance, with *n* ranging from 1 to 50 times. The number of features that were selected *n* times, as well as the expected number, for the functional atlas is shown in Figure 5A. The probability of a feature appearing ten times or more due to chance is very small. Therefore, we further analyzed all features that were selected 10 or more times resulting in four features.

We then asked how well we can classify a given dataset using only the best four features extracted from the functional atlas. We used the four features to classify our entire 170 subject set in a single cross validation scheme to produce a better estimate of classification power of these features. This resulted in a boost in classification performance from 64 to 78% (Figure 4B and Table 2), which was significantly above chance (*p* < 0.001).

**Table 2:** Performance summary.

Dataset				

Classifier	SVM			Adaboost
		
Dimensionality Reduction	SFS	LDA	Independent	SFS
**Prewhitened**				
Functional	**Acc. = 78% Sen. = 65%** Spe. = 65%	Acc. = 65%	Acc. = 65%	Acc. = 63% Sen. = 44% **Spe. = 85%**
AAL	**Acc. = 80% Sen. = 77%** Spe. = 68%	Acc. = 67%	Acc. = 73%	Acc. = 62% Sen. = 52% **Spe. = 80%**
**Raw**				
Functional	Acc. = 64%	Acc. = 63%		Acc. = 63%
AAL	Acc. = 64%	Acc. = 65%		Acc. = 61%


*Classification accuracy, sensitivity and specificity for different datasets, classifiers, dimensionality reduction methods, and atlases. SFS, Sequential Forward Selection; LDA, Linear Discriminant Analysis; Independent, Selecting top features based on their independent performance. Since classification performance using raw data was lower than that of prewhitened data using SFS and LDA feature selection methods, classification performance on raw data using the independent feature selection method is not reported for either atlas. Bold values represent the highest value obtained for each measure.*

The most frequent four features are listed in Table 3 with a description of the anatomical regions of the nodes involved and the corresponding network measure. These functional areas do not necessarily align with anatomical areas, therefore we report the names of the areas from the AAL atlas that had the highest overlap and percentage of the region overlap with that anatomical region. All four features captured the characteristics of pairs of nodes rather than single nodes or capturing global properties of the network as a whole. The single cross validation classification rate of each feature is also reported. While the best single feature achieved about 70% classification accuracy on its own, by combining them together the single cross validation performance was enhanced to 78% (Figure 4B). We then looked at frequency of each functional region showing up in the top 4 features. These four features were comprised of five functional regions. The location of the five nodes that had the highest frequency of appearing in the top four features are shown in Figure 5B. These anatomical regions (in order of highest frequency to lowest) were located in the left temporal lobe, right occipital lobe, central portion of bilateral thalami, and left frontal/parietal lobes. These four features included three networks measures: distance, generalized topological overlap, and matching index. Distribution of values of the most informative four features is shown in Supplementary Figure 6A.

**Table 3:** List of the most informative features.

Rank	Frequency of appearance (out of 50)	Name of network measure	First Anatomical Region with the highest overlap (% overlap)	Second Anatomical Region with the highest overlap (% overlap)	d’(*p*-value)	Single feature classification
** Functional atlas**					
1	21	Distance	Left Middle Temporal Gyrus(53), Left Superior temporal Pole(19)	Right Inferior Occipital Cortex(39), Right Lingual Gyrus(17)	0.87(<0.001)	69%
2	12	Generalized Topological Overlap	Left Middle Temporal Gyrus(53), Left Superior Temporal Pole(19)	Right Inferior Occipital Cortex(39), Right Lingual Gyrus(17)	-0.88(<0.001)	69%
3	10	Matching Index	Right Inferior Temporal Gyrus(50), Right Middle Temporal Gyrus(48)	Left Thalamus(49), Right Thalamus(43)	0.74(<0.001)	67%
4	10	Matching Index	Left Postcentral Gyrus(72), Left Precentral Gyrus(10)	Left Thalamus(49), Right Thalamus(43)	0.72(<0.001)	68%
** AAL**					
1	47	Matching Index	Left Postcentral Gyrus	Left Thalamus	0.94(<0.001)	73%


*From left, column 1: Rank of the feature in terms of frequency of appearing in the selected feature set; column 2: Number of appearances in the selected feature set; column 3: Name of network measure, all measures characterize relationship between pairs of nodes (regions); column 4: First region corresponding to the measure. Regions of the functional atlas do not have labels. To indicate their anatomical location, the two anatomical regions from the AAL atlas with the highest overlap are listed. The number in () shows the percent overlap; column 5: Same as column 2 for the second region corresponding to each region; 6th column: Sensitivity index, also known as *d’*and the 2 sample *t*-test *p*-value in () comparing distribution of each feature across the control and schizophrenic population. A positive *d’* value indicates that feature values are higher for the patients compared to control subjects; column 7: Performance of each feature on its own.*

Since the AAL atlas showed maximal accuracy with only a single feature, and adding more features was detrimental to classification performance for double cross validation, we focused on that single feature. This feature appeared in the top feature set 47 times out of 50 times, which was significantly above chance. This top feature was the matching index between left postcentral gyrus and left thalamus (Table 3). Location of these two regions is shown in Figure 5C. Distribution of values for this features is shown in Supplementary Figure 6B. This single feature was used to classify the entire subject set in a single cross validation scheme, which resulted in 80% classification accuracy (Figure 4C and Table 2), that is significantly above chance (*p* < 0.001).

We observed similar classification performances using both the functional and AAL atlases with other C values and kernels (Supplementary Figure 1). We also observed similar or less impressive results when we used the Adaboost classifier instead of SVM (Table 2 and Supplementary Figure 2).

We also looked at sensitivity and specificity of our classification algorithm (Supplementary Figure 3 and Table 2). Through the double cross validation scheme, sensitivity and specificity for the functional atlas were 65%. These values were higher for the AAL atlas with average sensitivity equal to 77% and specificity equal to 68%. Using the Adaboost classifier increased specificity and decreased sensitivity in both atlases. Maximum specificity obtained using the Adaboost was 85 and 80% for functional and AAL atlases respectively, whereas maximum sensitivity was 44 and 52% for functional and AAL atlases.

Next, we looked at different methods of dimensionality reduction (Supplementary Figure 4). We compared accuracy performance of the SFS algorithm with that of the Linear Discriminant Analysis (LDA) and independent method. The dimensionality reduction method did not affect performance accuracy (Table 2). However, both LDA and independent methods achieved the same performance using more features than the SFS algorithm.

We then analyzed the effect of prewhitening on performance accuracy (Table 2 and Supplementary Figure 5). Two functional atlases were constructed, one using prewhitened time series, and the other using the raw time series. These resulting functional atlases were then used to generate 90 time series from the prewhitened and raw data, from which graph measures were then quantified. Classification was then repeated on all four networks and classification accuracies were compared. We repeated the same procedure with the AAL atlas, when the atlas was applied to prewhitened and raw time series to construct the graph used for classification. We did not observe any difference between performance of the two functional atlases. However, classification performance when the AAL atlas was applied to prewhitened time series was 10% higher than that of raw time series.

**FIGURE 6:**
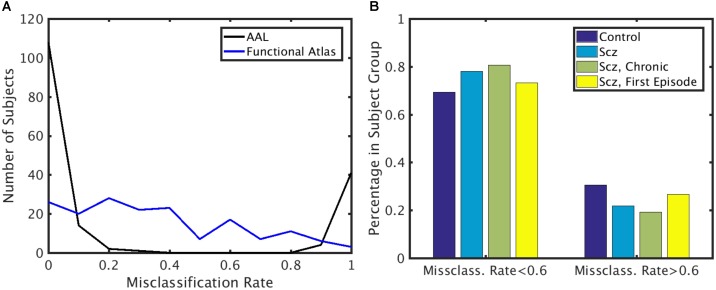
Misclassification rate. **(A)** Distribution of misclassification rate across subjects for the AAL and functional atlases. **(B)** Percentage of subjects in each subject group with low and high misclassification rate using the AAL atlas. *X*-axis: Subjects that are highly classifiable (misclassification rate < 0.6) and unclassifiable (misclassification rate > 0.6). *Y*-axis: Percentage of subjects in each subject group.

To identify if the misclassification of subjects could be attributed to disease class, we first calculated the misclassification rate for each subject for both functional and AAL atlases (Figure 6A) across all the folds. Misclassification rate for the functional atlas was fairly uniform. However, misclassification rate of the subjects using AAL atlas was bimodal. Some subjects were misclassified correctly more than 60% of the times, while others were misclassified less than 20% of the time. To understand the characteristics of the classifiable and unclassifiable group, we looked at the percentage misclassified in both the control and schizophrenic group (Figure 6B). We further looked at misclassification rate for the chronic and first episode schizophrenic patients groups. No trend for misclassification was observed in any of these classes, nor for age and gender (data not shown).

Another possible reason for misclassification of patients may be attributed to the severity of their symptoms, hypothesizing that mild subjects may have neural patterns similar to healthy subjects. To investigate this possibility we correlated the SANS and SAPS score of the patients with the misclassification rate (Supplementary Figure 7). We further correlated misclassification rates with each category of the SANS and SAPS scores with the misclassification rate. No significant correlation between any of the scores and misclassification rates were found.

**FIGURE 7:**
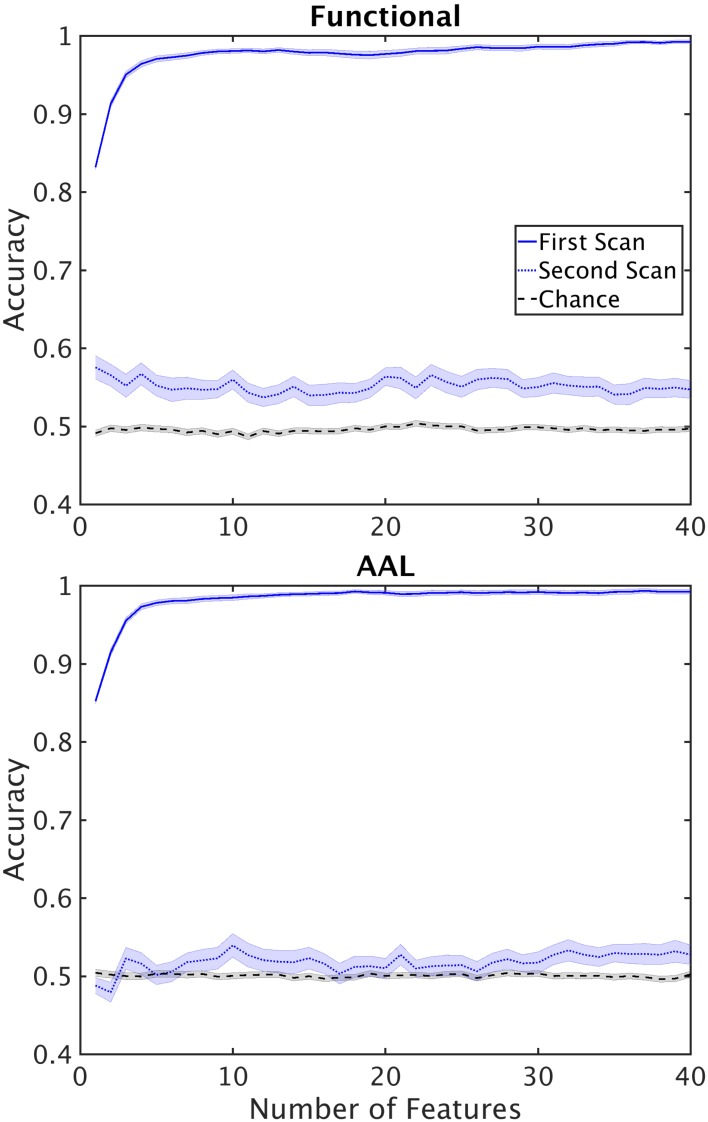
Performance of the classifier on second scans. Performance of the classifier when classifier optimization is optimized using the first scan and tested on second scans for functional **(top)** and AAL **(bottom)** atlases. Horizontal lines mark chance level.

Another finding we wished to explore more in depth was the difference in performance between the single and double cross validation. The drop in performance could be due to variability across subjects, or variability within subjects. Variability across subjects is caused by individual differences in the structure of functional networks. Within subject variability can be attributed to the different active networks observed during the scan resulting in different structure of the functional networks. To test how well reconstructed networks generalize within subjects we reconstructed networks from 42 subjects (24 control, 18 schizophrenic) for which we had two scans, taken 6 months apart. We used the dataset from the first scan for feature selection and classifier training. We then used the resultant classifier and feature set to classify the second dataset from the same subjects. This procedure tests the cross validation performance within subjects. Performance of the double cross validation across datasets is shown in Figure 7. The results show that variability in network structure constructed within patients is a significant contributor to poor generalizability of our classification.

## Discussion

In this study we developed and tested a classification pipeline to discriminate persons with schizophrenia from healthy controls. We used prewhitened BOLD time series to construct a network model of the brain, using both the AAL anatomical atlas and our functional atlas to define nodes of the network. We extracted multivariate graph theoretic measures and used them as features for classification of the subjects using linear SVM. Measures that were most informative to classification were identified as biomarkers for schizophrenia. We adopted a double cross validation scheme to identify the most informative features. The highest classification accuracy was 80% using the AAL atlas with a single feature: the matching index between left postcentral gyrus and left thalamus. Adding any other features decreased accuracy. Comparison of classification accuracy using the double cross validation scheme to single cross validation revealed that single cross validation results in inflated performance accuracies. Moreover, prewhitening of the time series significantly improves classification performance in double cross validation. A subset of the subjects, including both healthy and schizophrenic subjects, were misclassified more than 80% of the time. However, no significant correlation was found between misclassification rate of the patients and the severity of their symptoms.

We compared classification accuracy using AAL and the functional atlas to define the nodes used to construct the network model. Classification accuracy did not improve using a functional atlas over the AAL atlas. This may be because the duration of resting state activity was not long enough to robustly capture functional structure of the brain (Moghimi et al., 2017). Even though we obtained significantly above chance accuracy, the fairly high false positive and false negative rates indicates this method does not approach the necessary performance to be useful clinically.

Using machine learning techniques for biomarker identification using fMRI datasets has been extensively explored (see Zarogianni et al., 2013; Sundermann et al., 2014; Kambeitz et al., 2015). A summary of previous work in this area is provided in Supplementary Table 2. Our study builds upon existing work in the following aspects: (i) we used a large cohort of subjects, (ii) we performed double cross validation, (iii) we prewhitened the time series prior to construction of the network.

Machine learning techniques have also been used on other imaging modalities to identify biomarkers for schizophrenia (see Zarogianni et al., 2013; Kambeitz et al., 2015 for a review). Several studies have used structural T1 weighted MR images [e.g., (Iwabuchi et al., 2013), 77% accuracy, single cross validation, (Nieuwenhuis et al., 2012), 70.4%, double cross validation] and Diffusion Tensor Imaging (DTI) [e.g., (Ingalhalikar et al., 2010), 90.6% accuracy, using single cross validation].

The total number of features was an order of magnitude bigger than the number of subjects. To avoid the curse of dimensionality, we used the SFS algorithm (Guyon and Elisseeff, 2003) to reduce dimensionality of the dataset. We compared the results to two other dimensionality reduction methods, LDA and independent selection (Supplementary Figure 4). Both methods underperformed with respect to the SFS method. The SFS algorithm is more computationally expensive than the other two methods, but its major advantage is that it reduces the redundancy present in the dataset. More specifically, a fair level of correlation has been observed with network level characteristics of the brain (Lynall et al., 2010). Features that have correlation with each other, carry the same information, and are bound to have similar performances when used independently. But their combination does not result in higher performance due to the correlation. Therefore, the use of more complex feature selection methods, despite their upfront computational expense, may result in better classification with less computational effort for classification.

The considerable degradation of performance from single to double cross validation demonstrates the importance of testing the final performance on out of sample data, excluding data that was used in feature selection using single cross validation results. Results reported based on single cross validation are overly optimistic for out of sample data (Sundermann et al., 2014). In fact, simulations have shown that even when two classes of data points are generated from the same distribution (i.e., there is no meaningful difference between the two classes), single cross validation is biased toward above chance classification accuracy (Simon et al., 2003). Double cross validation prevents the classifier from overfitting to the dataset that is used for biomarker discovery. Double cross validation has been employed in several classification studies including schizophrenia using fMRI (see Supplementary Table 2), schizophrenia using T1 weighted structural images (Nieuwenhuis et al., 2012; Koutsouleris et al., 2015), major depressive disorder using fMRI (Rosa et al., 2015), and autism using fMRI (Anderson et al., 2011). However, the majority of studies do not report double cross validation results (Supplementary Table 2), presumably due to limited sample size (Sundermann et al., 2014). Our results, directly comparing single and double classification performances, supports our hypothesis that single cross validation reports overly inflated accuracy rates. As previously suggested by others in brain imaging (Sundermann et al., 2014) and genetic (Simon et al., 2003) biomarker identification fields, we propose adoption of double cross validation as a standard paradigm for biomarker discovery using brain imaging datasets.

We also performed double cross validation across datasets taken from the same subject but at different times (Figure 7). We found that within subject classification rate was similar to the between subject classification rate for double cross validation results. This shows that inherent variability in fMRI datasets caused by the state of the subject during the scan poses a serious challenge in generalizability of the results. An important remedy to this problem is longer scan durations, or multiple scans across several sessions. Typical scan duration for classification studies of schizophrenia has been between 5 and 10 min (Supplementary Table 2) and may be insufficiently long to fully characterize network connectivity.

In addition, we explored the effect of the atlas used to define nodes on the classification performance by comparing the AAL anatomical atlas with a functional atlas constructed from our dataset. The functional atlas was constructed using data from our control subjects only which introduces a bias in structure of the atlas. However, we excluded the patient group from construction of the atlas because schizophrenia alters structural and functional connectivity pattern of the brain (Camchong et al., 2011). Moreover, group functional atlases such as ours capture functional organization of the brain shared by all the subjects in the group and are robust to idiosyncrasies unique to each subject (Gordon et al., 2014). Despite our hopes that a functional atlas would boost performance, we found the classification accuracy using the AAL atlas was higher. This observation does not necessarily mean that anatomical atlases are superior to functional atlases. Extensive evaluation of our functional parcellation algorithm concluded that our dataset was not long enough for construction of a robust functional atlas (Moghimi et al., 2017). Previous studies have concluded that minimum duration of resting state activity required for construction of a functional atlas that is replicable across different datasets from the same group of subjects is approximately 27 min (Laumann et al., 2015), which is more than four times the duration we used (6 min) for construction of the functional atlas. While the use of anatomical atlases for classification remains the norm, a few studies have used atlases constructed using fMRI (Arbabshirani et al., 2013, 2014a; Rashid et al., 2016; Singh and Bagler, 2016) or diffusion tensor imaging (DTI) data (Hu X. et al., 2013; Wang et al., 2016). One study observed a ∼10% increase in single cross validation accuracy with a data driven atlas constructed using DTI data over an anatomical atlas (Wang et al., 2016). However, we observed a 2% decrease in double cross validation accuracy using our functional atlas compared to the anatomical atlas. Several studies have used Independent Component Analysis (ICA) to produce parcellations from resting state fMRI activity (Arbabshirani et al., 2013, 2014a; Rashid et al., 2016; Singh and Bagler, 2016) (Supplementary Table 2). ICA does not produce contiguous regions, rather functional networks, comprising multiple regions. A parcellation with contiguous regions makes it easier to localize the biomarker to a brain region that is impacted by the disorder. If a single region within a functional network is implicated in the disease, the entire network will be implicated using a network based parcellation, which includes regions that are not affected by the disease.

We used multivariate network level measures as classification features in this study, including a mixture of global measures as well as measures that characterize single regions or pairwise statistics. The feature set extracted from resting state fMRI for classification varies widely across studies. One common feature is the pairwise correlation coefficient between average time series from different brain regions (Tang et al., 2012; Venkataraman et al., 2012; Guo et al., 2013; Su et al., 2013; Yu et al., 2013; Shen et al., 2014; Kim et al., 2016). This bivariate feature, however, fails to pick up on more sophisticated motifs in the functional structure of the brain. Network measures, being multivariate, are capable of identifying more complex patterns in group differences and have been used in several classification studies (Bassett et al., 2012; Anderson and Cohen, 2013; Fekete et al., 2013; Singh and Bagler, 2016). However these studies either use global networks measures (Bassett et al., 2012; Anderson and Cohen, 2013; Fekete et al., 2013), or use average and standard deviation of local measures (Singh and Bagler, 2016), which eliminates spatial information about the most discriminating regions. Our data driven greedy feature selection method preserves the identity of the nodes and links that are most informative for classification.

Our highest classification accuracy was achieved with a single network measure based on the AAL atlas. As more features were used for classification, single cross validation accuracy increased but double cross validation accuracy decreased. This indicates that the added features did not generalize well across subjects; their addition to the feature set caused the classifier to weight on other features, diluting useful information. Using the functional atlas, we found four features whose appearance in the selected feature set was statistically meaningful. The reduction from a 19000+ feature space to a few features, reveals the tremendous redundancy inherent to the dataset. Similar to our results, (Fan et al., 2011) obtained a 85% double cross validation accuracy using seven features. In another study, (Tang et al., 2012) obtained 93.2% accuracy double cross validation accuracy using 550 features.

Prewhitening of the time series increased classification performance in this study. A similar observation was made using fMRI to classify PTSD patients from controls (Christova et al., 2015). In contrast to our results, (Arbabshirani et al., 2014b) did not observe any difference between discriminability of prewhitened and raw time series in a cohort of healthy subjects and subjects with schizophrenia. However, the (Arbabshirani et al., 2014b) study compared bivariate measures across the groups, whereas in our study we used multivariate measures, which may detect more complex features that distinguish the groups.

Of the 25 different network measures used to generate features (listed in the Supplementary Table 1), the five most informative features came from three measures: distance, generalized topological overlap, and matching index. All of the top five features were from pairwise network measures. We did not find network or nodal metrics that provided significant classification information. Each measure and the observed trends associated with them are discussed more in detail.

The single feature that produced maximum classification accuracy using the AAL atlas was an increase in matching index between the postcentral gyrus and left thalamus in schizophrenic patients (Table 3 and Supplementary Figure 6). Matching index between two nodes quantifies the similarity between their functional connectivity profiles based on the number of common neighbors between the two nodes and is applicable to binary graphs (Rubinov and Sporns, 2010b). Postcentral gyrus has been implicated in schizophrenia in several other studies (Yang et al., 2010; Castro et al., 2011; Rashid et al., 2016). Interestingly, another study reported that functional connectivity between the left postcentral gyrus and right thalamus was different across the healthy and schizophrenic group (Kim et al., 2016).

The first feature selected by the functional atlas was the distance between two regions in the left temporal and right occipital lobes. Distance between two nodes is the shortest path between them in a binary graph (Rubinov and Sporns, 2010b). As reported in Table 3 and Supplementary Figure 6, the distance between two regions in the right occipital lobe and left temporal lobe is lower in the control group compared to schizophrenic patients. Interestingly, the distance between these two nodes in the majority of control subjects is 1, meaning that the two regions are connected to each other directly via a single link, while the distance between these same two nodes is 2 in the majority of schizophrenic subjects, which means the direct link between the two nodes is absent in patient group resulting in hypoconnectivity between these two regions. Changes in volume of the left middle temporal gyrus in schizophrenic patients has previously been reported (Onitsuka et al., 2004; Hu M. et al., 2013). Moreover, the middle temporal gyrus has been implicated in other fMRI classification studies (Yang et al., 2010; Castro et al., 2011), albeit bilaterally. Disruption in functional activity of the right inferior occipital gyrus has been reported in another study (Castro et al., 2011).

The second most informative feature generated using the functional atlas was the generalized topological overlap between the same two regions (Table 3). Generalized topological overlap quantifies the extent to which a pair of nodes have similar *m*-th step neighbors in binary graphs (Rubinov and Sporns, 2010b). The *m*-th step neighbors of a node are all the nodes in the binary graph that are reachable through a maximum of *m* steps. We observed that generalized topological overlap between regions in the right occipital lobe and left temporal lobe is higher in the control group compared to patients (Supplementary Figure 6). This shows that functional connectivity pattern between these two regions diverges from each other in the patient group.

The third most informative feature using the functional atlas was the matching index between two regions in the right temporal lobe and the thalamai (Table 3). We observed increased matching index between regions in the right temporal gyrus and bilateral thalami in the schizophrenic group, when compared to controls (Table 3 and Supplementary Figure 6). The first region overlapped with the right inferior and middle temporal gyri. The other region overlapped with the ventral portion of bilateral thalami. As mentioned earlier, middle temporal gyrus has been implicated in schizophrenia in other classification studies (Yang et al., 2010; Castro et al., 2011). Disruption of functional connectivity of thalamus has also been reported in several other studies (Skudlarski et al., 2010; Atluri et al., 2015; Kim et al., 2016).

Increased matching index in the schizophrenic group was also observed between another pair of regions, the fourth most informative measure using functional atlas (Table 3 and Supplementary Figure 6). The first region overlapped with both postcentral and precentral gyri, and the second region overlapped with the ventral portion of bilateral thalami. This feature is highly similar to the most informative feature found in the AAL atlas. The fact that this feature shows up as a top feature regardless of the atlas is in some way a validation of the feature.

Several improvements could increase classification performance necessary to approach clinically useful accuracy. First, acquiring longer durations of functional activity results in more robust functional networks that can enhance performance, especially decreasing the gap between single and double cross validation results. The second improvement may be to use more robust brain atlases. Anatomical atlases are based on physical landmarks of the brain, resulting in brain regions that potentially encompass several histologically distinct regions with distinct functional roles. Functional atlases can be constructed at finer granularity levels. However, our functional atlas was constructed using 6 min of resting state activity, which might not be enough to capture functional organization of the brain. Recent effort to construct brain atlases using multi-modal datasets such as combining resting and task fMRI with myelin maps (Glasser et al., 2016) are promising. Third, more robust biomarkers can be developed by using of multi-modal feature sets, by combining feature extracted from different modalities such as T1 weighted images, fMRI, and DTI (e.g., Silva et al., 2014). The feature set can further be supplemented with non-brain related datasets such as genetic biomarkers (e.g., Yang et al., 2010). Fourth, medication load could be a confounding factor that we could not adequately account for. Unfortunately, the study of unmedicated schizophrenic patients is very challenging but could provide useful information.

## Conclusion

Biomarker identification using classification algorithms requires double cross validation to increase robustness of the results. Without proper cross validation, reported classification power is inflated and the identified biomarkers are poorly generalizable to new subject groups. Multivariate network measures are a promising set of features for biomarker identification. Using these measures, a functional atlas based on the short resting state recordings did not provide a parcellation that improved classification over the anatomical atlas. In addition, prewhitening of the fMRI time series when calculating interactions between brain areas separates changes in the dynamics of brain areas from changes in interactions, improving network measures. However, training on one set of scans and classifying on another set of scans from the same subjects resulted in classification rates similar to across subject classification, indicating that subject state during the short scan may significantly limit classification accuracy.

## Ethics Statement

This study was carried out in accordance with the recommendations of University of Minnesota’s Institutional Review Board with written informed consent from all subjects. All subjects gave written informed consent in accordance with the Declaration of Helsinki. The protocol was approved by the University of Minnesota’s Institutional Review Board.

## Author Contributions

PM, KL, and TN designed the experiment and wrote the manuscript. PM conducted the experiment.

## Conflict of Interest Statement

The authors declare that the research was conducted in the absence of any commercial or financial relationships that could be construed as a potential conflict of interest.

## Footnotes

1 https://fsl.fmrib.ox.ac.uk/fsl/fslwiki/MELODIC
