# Supplementary material for: Data Driven Classification Using fMRI Network Measures: Application to Schizophrenia
Source: Front Neuroinform. 2018 Oct 30;12:71. doi: 10.3389/fninf.2018.00071 (PMC6218612; doi:10.3389/fninf.2018.00071)
Supplement: Supplementary file 1 [file Presentation_1.pdf]

Supplementary Material

We used multivariate network level measures extracted from 6 minute resting state fMRI activity of a cohort of 170 healthy and schizophrenic subjects. All time series were prewhitened prior to calculating the network measures. Nodes of the network were either defined by the AAL anatomical atlas, or functional atlas constructed using fMRI activity itself. The measures (Supplementary Table 1) were used as features to classify the subjects into healthy or schizophrenic using support vector machines (SVM). Prior to classification, the sequential forward selection (SFS) method was used to select features that were the most informative about the state of each subject. We adopted a double cross validation scheme to select such features and report an unbiased estimate of classification accuracy.

We tested several values for the box-constraint (C value) and kernel of the SVM. We used C values equal to 0.1, 1, and 10. We also tested linear, quadratic, polynomial (degree=3), and radial basis function (RBF, sigma = 1) kernels (Supplementary Figure 1). In addition to SVM, we also report classification performance using an Adaboost classification approach, by training 10 weak SVM classifiers (Supplementary Figure 2). Classification Sensitivity and Specificity for both SVM and Adaboost classifiers are also reported (Supplementary Figure 3).

In addition to the SFS feature selection method, we used two other feature selection methods: Linear Discriminant Analysis (LDA) and the independent method where top single features are selected based on their classification accuracy when used independently for classification. Classification performance of these feature selection methods are compared in Supplementary Figure 4.

To investigate the effect prewhitening has on classification performance, we also calculated the network measures using the raw time series, without removing autocorrelation, and compared the resultant classification accuracy to that of prewhitened time series. The functional atlas was also constructed using raw time series. The results are shown in Supplementary Figure 5.

Once the most informative features were selected using the SFS method, we looked at distribution of values for each feature across the healthy and schizophrenic groups. We also collapsed the schizophrenic group into first episode and chronic groups to see whether there is a difference between the two schizophrenic groups. The results are shown in Supplementary Figure 6.

Finally, we inspected the relationship between severity of the disease, as quantified by SANS and SAPS scores, and misclassification rate for each of our schizophrenic subjects (Supplementary Figure 7).

Supplementary Tables

Supplementary Table 1

Measure Number	Measure Name	Graph type	Measure type	Extra Parameters
1	Number of Communities	Weighted	Global	We used the Louvain community detection algorithm.
2	Average Community Size	Weighted	Global	
3	Transitivity	Weighted	Global	
4	Assortativity	Weighted	Global	
5	Rich Club Curve	Binary	Global	Calculated for a range of degrees, from 1 to the average node degree of the network .
6	Characteristic Path Length	Binary	Global	
7	Global Efficiency	Weighted	Global	
8	Radius of Graph	Binary	Global	
9	Diameter of graph	Binary	Global	
10	Node Betweenness Centrality	Weighted	Node	
11	Eigenvector Centrality	Weighted	Node	
12	Shannon Entropy	Weighted	Node	
13	Within Module Degree Centrality	Weighted	Node	
14	Participation Coefficient	Weighted	Node	

15	Clustering Coefficient	Weighted	Node	
16	Node Degree	Weighted	Node	
17	Strength	Weighted	Node	
18	Eccentricity	Binary	Node	
19	Local Efficiency	Weighted	Node	
20	Reachability	Binary	Pair	
21	Distance	Binary	Pair	
22	Weighted Distance	Weighted	Pair	
23	Overlap Amongst Neighbors	Weighted	Pair	
24	Generalized Topological Overlap	Binary	Pair	Calculated up to 3rd step neighbors.
25	Matching Index	Binary	Pair	

Supplementary Table 1. List of graph theoretic measures used for classification. From left to right: column 1, measure number; 2, measure name (Bullmore and Sporns 2009; Rubinov and Sporns 2010); column 3, type of graph used for calculating the measure. Some measures are specific to binary graphs. To calculate these measures the weighted graph was thresholded and converted to binary; column 4, type of measure. For a graph with N nodes, global measures characterize the entire network and produce one value. Measure that characterize nodes produce N values. Measure that characterize measure pairs produce $N.(N-1)/2$values; column 5, Some measure require extra parameters to be calculated. Value of the parameters are specified in this column.

References

Bullmore, E., and Sporns, O. (2009). Complex brain networks: graph theoretical analysis of structural and functional systems. Nat. Rev. Neurosci. 10, 186–198. doi: 10.1038/nrn2575

Rubinov, M., and Sporns, O. (2010). Complex network measures of brain connectivity: uses and interpretations. Neuroimage 52, 1059–1069. doi: 10.1016/ j.neuroimage.2009.10.003

Supplementary Table 2

Dataset	Number of Subjects	Features	Dimensionality Reduction Method(s)	Classifier(s)	Double Cross Validation	Classification Accuracy	Reference
fMRI, Oddball Auditory Task	68, Scz = 34, H = 34	Activation Map	LDA + mRMR	SVM	No	88%	(Juneja, Rana, and Agrawal 2014)
fMRI, Category Exemplar Word Pair Task	25, Scz = 15, H = 10	Activation Map	PCA	LDA	No	>80%	(Ford et al. 2003)
fMRI, AX-Continuous performance test	102, Scz = 51, H = 51	Behavioral Performance	None	LDA	No	58%	(Yoon et al. 2012)
		Activation contrast between A and B cues in DLPFC				62%	
		Activation contrast between A and B cues in the entire brain				59%	
fMRI, resting state, 6 minutes	83, Scz = 48, H = 35	Regional Homogeneity, regions defined anatomically	PCA	LDA	No	80%	(Shi et al. 2007)
		Voxelwise homogeneity				74%	
fMRI, Resting State, 6 minutes	52, Scz = 32, H = 20	Pairwise Correlation Coefficient between anatomically defined regions	Kendall tau rank correlation coefficient feature selection + locally linear embedding manifold learning	K-means clustering	No	87%	(Shen et al. 2010)
fMRI, Auditory Oddball task and Genetic data	40, Scz = 20, H = 20	Genetic data, Activation map and ICA map	Forward Sequential feature selection method	SVM	No	87%	(Yang et al. 2010)

fMRI, verbal fluency task	104, Scz = 32, H = 40, Bipolar = 32	Activation map	None	SVM, three way classification	No	92%, Scz vs non-Scz	(Costafreda et al. 2011)
fMRI, resting state, 6 minutes	58, Scz = 29, H = 29	Size of the largest connected component	None	SVM	No	75%	(Bassett et al. 2012)
fMRI, resting state	68, Svz = 40, H = 28	Lattice Auto-Associative Memories	None	k-NN	No	~83%	(Chyzhyk and Graña 2015)
fMRI, resting state, >10 minutes	36, Scz = 18, H = 18	Pairwise regional functional connectivity between anatomically defined regions	Decision tree feature selection	Decision tree	No	75%	(Venkataraman et al. 2012)
fMRI, resting state, 6 minutes	146, Scz = 72, H = 74	Voxel-wise regional homogeneity, amplitude of low fluctuations, and functional homotopy	Feature selection based on class distance	SVM and random forests	No	80%	(Savio and Graña 2015)
fMRI, resting state, 10 minutes	20, Scz = 10, H = 10	Fine Granularity Functional Interaction between subnetworks	Separability threshold + PCA	SVM	No	77.5%	(Hu et al. 2013)
		Functional connectivity				77.5%	
		Fine Granularity Functional Interaction between subnetworks + Functional connectivity				95%	
fMRI, resting state, 6 minutes	71, Scz = 24, H = 22, Healthy Siblings of Scz subjects = 25	Pairwise Correlation Coefficient between anatomically defined regions	PCA	SVM, 3 way classification between Schizophrenic patients, their healthy siblings, and healthy controls	No	62%	(Yu et al. 2013)
fMRI, resting state, >5	370, Scz = 195, H	Pairwise Correlation	mRMR	SVM	Yes	84%	(Arbabshirani, Castro, and

minutes	= 175	Coefficient between networks defined by ICA					Calhoun 2014)
		Auto connectivity of network time series				80%	
		Pairwise Correlation Coefficient between networks defined by ICA +auto connectivity of network time series				88%	
fMRI, 0- and 2-back memory task	37, Scz = 17, H = 20	Activation Map	Searchlight Based feature Extraction	SVM	Yes	91	(Bleich-Cohen et al. 2014)
fMRI, resting state, 6 minutes	64, Scz = 32, H = 32	Pairwise Pearson Correlation Coefficient between anatomically defined regions	Thresholded Kendall-Tau coefficient	SVM, linear	No	81.2%	(Su et al. 2013)
		Maximal Information Coefficient				76.6%	
		Extended Maximal Information Coefficient				82.8%	
fMRI, resting state, 6 minutes	49, Scz = 24, H = 25	Pairwise Correlation Coefficient Over Time between Regions of interest determined by Task Activity	Threshold based on t-test	SVM, Polynomial Kernel	Yes	81.3%	(Shen et al. 2014)
				SVM, Linear		73.5%	
				SVM, Gaussian		79.6%	
fMRI, resting state, 5 minutes	100, Scz = 50, H = 50	Pairwise Correlation Coefficient between anatomically defined Regions	None	Deep Neural Network	Yes	85%	(Kim et al. 2016)
				SVM, Linear		77%	
fMRI, resting	18, Scz =	Network Level	Threshold	SVM, Block	Yes	96%	(Fekete et al.

state, 10 minutes	8, H = 10	Measures using nodes defined by the AAL atlas, constructed multiple graphs	based on t-test + Recursive Feature Elimination	Diagonal Optimization with Spherical Kernels			2013)
fMRI, resting state, 5 minutes	56, Scz = 28, H = 28	Z-map of spatial components identified using ICA	T-test + PCA + LDA	Nearest Neighbors	No	93%	(Du et al. 2012)
fMRI, Auditory Oddball task						98%	
fMRI, resting state, 6 minutes	146, Scz = 72, H = 74	Network measures based on networks identified by ICA	None	SVM, Radial basis kernel	No	65%	(Anderson and Cohen 2013)
fMRI, resting state, 5 minutes	56, Scz = 28, H = 28	Functional connectivity between networks identified by ICA	None	SVM, linear	No	83%	(Arbabshirani et al. 2013)
				SVM, Radial basis and polynomial kernels		96%	
				Decision trees		96%	
				K nearest neighbor classifier		96%	
fMRI, resting state, >5 minutes	159, Scz = 60, H = 61, Bipolar = 38	Functional connectivity between networks identified by ICA	Double input symmetric relevance (DISR)	Three-way, SVM, linear	Yes	59%	(Rashid et al. 2016)
		Functional connectivity between networks identified by ICA over time				84%	
		Combination of the above feature sets				89%	
fMRI, Auditory oddball task	52, Scz = 31, H = 21	Gramian between regions identified using ICA, using	Recursive Feature Elimination	Multiple Kernel Learning	Yes	85%	(Castro et al. 2011)

		magnitude and phase information separately					
fMRI, Monetary Incentive Delay Task	98, Scz = 44, H = 54	Activation Map	None	SVM	No	85%	(Koch et al. 2015)
fMRI, resting state, 6 minutes	146, Scz = 72, H = 74	Binary and weighted network measures based on regions identified by ICA	All possible single, pair, triads and tetrads of features	SVM	No	65%	(Singh and Bagler 2016)
fMRI, Auditory odd ball task	70, Scz = 34, H = 36	Activation map	ICA + PCA	Projection pursuit algorithm	No	91%	(Demirci, Clark, and Calhoun 2008)
fMRI, resting state, 6 minutes	62, Scz = 31, H = 31	Functional connectivity pattern identified by ICA	Sequential forward selection feature selection	SVM	Yes	85.5%	(Fan et al. 2011)
fMRI, resting state, 6 minutes	44, Scz = 22, H = 22	Pairwise correlation coefficient between anatomically defined regions	Kendall tau rank correlation coefficient + PCA	SVM	Yes	93.2%	(Tang et al. 2012)
fMRI, resting state, 6 minutes	274, Scz = 152, H = 122	Z-scored pairwise correlation coefficient between specific anatomically delineated regions	None	SVM, Radial basis function kernel	No	73.4%	(Guo et al. 2013)

Supplementary Table 2. Summary of Previous Work. A summary of other classification studies of schizophrenia using fMRI datasets. This list is not exhaustive, but a limited survey of literature.

References

Anderson, Ariana, and Mark S. Cohen. 2013. “Decreased Small-World Functional Network Connectivity and Clustering across Resting State Networks in Schizophrenia: An fMRI Classification Tutorial.” *Frontiers in Human Neuroscience.* 7:520.

Arbabshirani, Mohammad R., Eduardo Castro, and Vince D. Calhoun. 2014. “Accurate Classification of Schizophrenia Patients Based on Novel Resting-State fMRI Features.” *Conference Proceedings: Annual International Conference of the IEEE Engineering in Medicine and Biology Society*:6691–94.

Arbabshirani, Mohammad R., Kent A. Kiehl, Godfrey D. Pearlson, and Vince D. Calhoun. 2013. “Classification of Schizophrenia Patients Based on Resting-State Functional Network Connectivity.” *Frontiers in Neuroscience* 7:133.

Bassett, Danielle S., Brent G. Nelson, Bryon A. Mueller, Jazmin Camchong, and Kelvin O. Lim. 2012. “Altered Resting State Complexity in Schizophrenia.” *NeuroImage* 59(3):2196–2207.

Bleich-Cohen, Maya, Shahar Jamshy, Haggai Sharon, Ronit Weizman, Nathan Intrator, Michael Poyurovsky, and Talma Hendler. 2014. “Machine Learning fMRI Classifier Delineates Subgroups of Schizophrenia Patients.” *Schizophrenia Research* 160(1-3):196–200.

Bullmore, Ed, and Olaf Sporns. 2009. “Complex Brain Networks: Graph Theoretical Analysis of Structural and Functional Systems.” *Nature Reviews. Neuroscience* 10(3):186–98.

Castro, Eduardo, Manel Martínez-Ramón, Godfrey Pearlson, Jing Sui, and Vince D. Calhoun. 2011. “Characterization of Groups Using Composite Kernels and Multi-Source fMRI Analysis Data: Application to Schizophrenia.” *NeuroImage* 58(2):526–36.

Chyzhyk, Darya, and Manuel Graña. 2015. “Classification of Schizophrenia Patients on Lattice Computing Resting-State fMRI Features.” *Neurocomputing* 151, Part 1:151–60.

Costafreda, Sergi G., Cynthia H. Y. Fu, Marco Picchioni, Timothea Toulopoulou, Colm McDonald, Eugenia Kravariti, Muriel Walshe, Diana Prata, Robin M. Murray, and Philip K. McGuire. 2011. “Pattern of Neural Responses to Verbal Fluency Shows Diagnostic Specificity for Schizophrenia and Bipolar Disorder.” *BMC Psychiatry* 11(1):18.

Demirci, Oguz, Vincent P. Clark, and Vince D. Calhoun. 2008. “A Projection Pursuit Algorithm to Classify Individuals Using fMRI Data: Application to Schizophrenia.” *NeuroImage* 39(4):1774–82.

Du, Wei, Vince D. Calhoun, Hualiang Li, Sai Ma, Tom Eichele, Kent A. Kiehl, Godfrey D. Pearlson, and Tülay Adali. 2012. “High Classification Accuracy for Schizophrenia with Rest and Task FMRI Data.” *Frontiers in Human Neuroscience* 6:145.

Fan, Yong, Yong Liu, Hong Wu, Yihui Hao, Haihong Liu, Zhening Liu, and Tianzi Jiang. 2011. “Discriminant Analysis of Functional Connectivity Patterns on Grassmann Manifold.” *NeuroImage* 56(4):2058–67.

Fekete, Tomer, Meytal Wilf, Denis Rubin, Shimon Edelman, Rafael Malach, and Lilianne R. Mujica-Parodi. 2013. “Combining Classification with fMRI-Derived Complex Network Measures for Potential Neurodiagnostics.” *PloS One* 8(5):e62867.

Ford, James, Hany Farid, Fillia Makedon, Laura A. Flashman, Thomas W. McAllister, Vasilis Megalooikonomou, and Andrew J. Saykin. 2003. “Patient Classification of fMRI Activation Maps.” In *Medical Image Computing and Computer-Assisted Intervention - MICCAI 2003*,

58–65. Lecture Notes in Computer Science. Springer, Berlin, Heidelberg.
Guo, Shuixia, Keith M. Kendrick, Jie Zhang, Matthew Broome, Rongjun Yu, Zhening Liu, and Jianfeng Feng. 2013. “Brain-Wide Functional Inter-Hemispheric Disconnection Is a Potential Biomarker for Schizophrenia and Distinguishes It from Depression.” *NeuroImage. Clinical* 2:818–26.
Hu, Xintao, Dajiang Zhu, Peili Lv, Kaiming Li, Junwei Han, Lihong Wang, Dinggang Shen, Lei Guo, and Tianming Liu. 2013. “Fine-Granularity Functional Interaction Signatures for Characterization of Brain Conditions.” *Neuroinformatics* 11(3):301–17.
Juneja, A., B. Rana, and R. K. Agrawal. 2014. “A Novel Approach for Classification of Schizophrenia Patients and Healthy Subjects Using Auditory Oddball Functional MRI.” In *Artificial Intelligence (MICAI), 2014 13th Mexican International Conference on*, 75–81.
Kim, Junghoe, Vince D. Calhoun, Eunsoo Shim, and Jong-Hwan Lee. 2016. “Deep Neural Network with Weight Sparsity Control and Pre-Training Extracts Hierarchical Features and Enhances Classification Performance: Evidence from Whole-Brain Resting-State Functional Connectivity Patterns of Schizophrenia.” *NeuroImage* 124:127–46.
Koch, Stefan P., Claudia Hägele, John-Dylan Haynes, Andreas Heinz, Florian Schlagenhauf, and Philipp Sterzer. 2015. “Diagnostic Classification of Schizophrenia Patients on the Basis of Regional Reward-Related FMRI Signal Patterns.” *PloS One* 10(3):e0119089.
Rashid, Barnaly, Mohammad R. Arbabshirani, Eswar Damaraju, Mustafa S. Cetin, Robyn Miller, Godfrey D. Pearlson, and Vince D. Calhoun. 2016. “Classification of Schizophrenia and Bipolar Patients Using Static and Dynamic Resting-State fMRI Brain Connectivity.” *NeuroImage* 134:645–57.
Rubinov, Mikail, and Olaf Sporns. 2010. “Complex Network Measures of Brain Connectivity: Uses and Interpretations.” *NeuroImage* 52(3):1059–69.
Savio, Alexandre, and Manuel Graña. 2015. “Local Activity Features for Computer Aided Diagnosis of Schizophrenia on Resting-State fMRI.” *Neurocomputing* 164:154–61.
Shen, Hui, Zhenfeng Li, Ling-Li Zeng, Lin Yuan, Fanglin Chen, Zhening Liu, and Dewen Hu. 2014. “Internetwork Dynamic Connectivity Effectively Differentiates Schizophrenic Patients from Healthy Controls.” *Neuroreport* 25(17):1344–49.
Shen, Hui, Lubin Wang, Yadong Liu, and Dewen Hu. 2010. “Discriminative Analysis of Resting-State Functional Connectivity Patterns of Schizophrenia Using Low Dimensional Embedding of fMRI.” *NeuroImage* 49(4):3110–21.
Shi, Feng, Yong Liu, Tianzi Jiang, Yuan Zhou, Wanlin Zhu, Jiefeng Jiang, Haihong Liu, and Zhening Liu. 2007. “Regional Homogeneity and Anatomical Parcellation for fMRI Image Classification: Application to Schizophrenia and Normal Controls.” *Medical Image Computing and Computer-Assisted Intervention: International Conference on Medical Image Computing and Computer-Assisted Intervention* 10 (Pt 2):136–43.
Singh, Megha, and Ganesh Bagler. 2016. “Network Biomarkers of Schizophrenia by Graph Theoretical Investigations of Brain Functional Networks.” *arXiv [q-bio.QM]*. http://arxiv.org/abs/1602.01191.
Su, Longfei, Lubin Wang, Hui Shen, Guiyu Feng, and Dewen Hu. 2013. “Discriminative Analysis of Non-Linear Brain Connectivity in Schizophrenia: An fMRI Study.” *Frontiers in Human Neuroscience* 7:702.
Tang, Yan, Lifeng Wang, Fang Cao, and Liwen Tan. 2012. “Identify Schizophrenia Using Resting-State Functional Connectivity: An Exploratory Research and Analysis.” *Biomedical Engineering Online* 11:50.
Venkataraman, Archana, Thomas J. Whitford, Carl-Fredrik Westin, Polina Golland, and Marek Kubicki. 2012. “Whole Brain Resting State Functional Connectivity Abnormalities in Schizophrenia.” *Schizophrenia Research* 139 (1-3):7–12.
Yang, Honghui, Jingyu Liu, Jing Sui, Godfrey Pearlson, and Vince D. Calhoun. 2010. “A Hybrid Machine Learning Method for Fusing fMRI and Genetic Data: Combining Both Improves

Classification of Schizophrenia." *Frontiers in Human Neuroscience* 4:192.
Yoon, Jong H., Danh V. Nguyen, Lindsey M. McVay, Paul Deramo, Michael J. Minzenberg, J. Daniel Ragland, Tara Niendham, Marjorie Solomon, and Cameron S. Carter. 2012. "Automated Classification of fMRI during Cognitive Control Identifies More Severely Disorganized Subjects with Schizophrenia." *Schizophrenia Research* 135 (1-3):28–33.
Yu, Yang, Hui Shen, Huiran Zhang, Ling-Li Zeng, Zhimin Xue, and Dewen Hu. 2013. "Functional Connectivity-Based Signatures of Schizophrenia Revealed by Multiclass Pattern Analysis of Resting-State fMRI from Schizophrenic Patients and Their Healthy Siblings." *Biomedical Engineering Online* 12(10).

Supplementary Figures

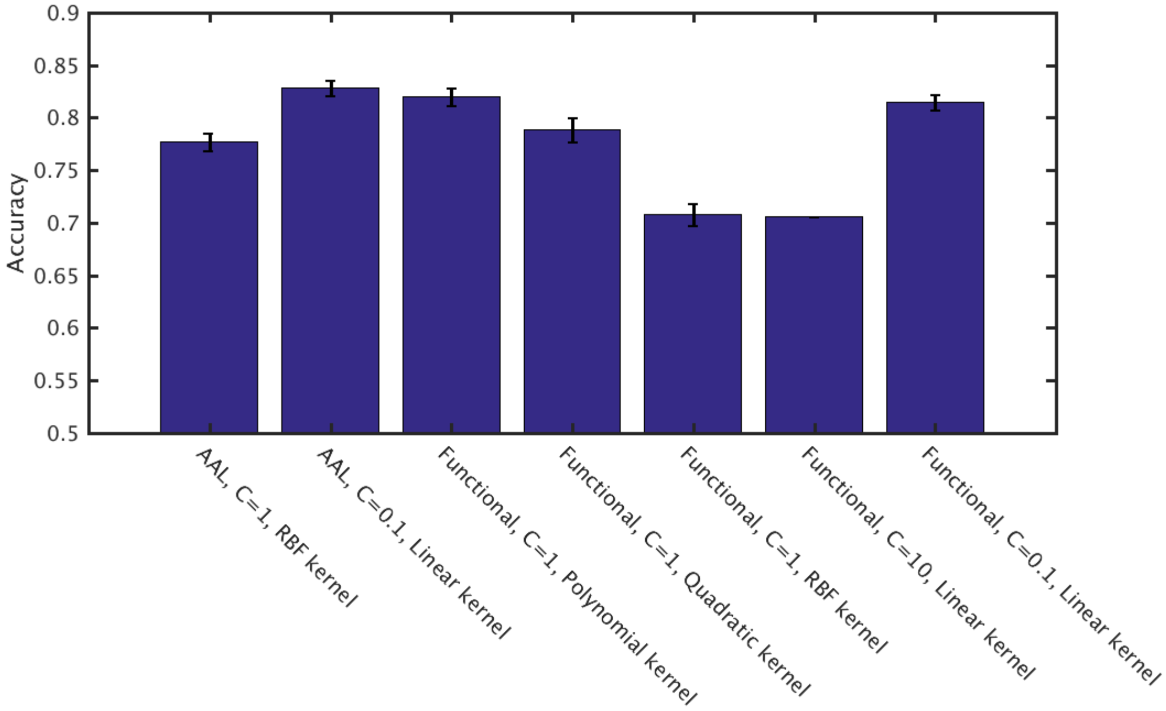

Supplementary Figure 1. Performance using different parameters
Classification accuracy when other C values or kernels were used for classification.

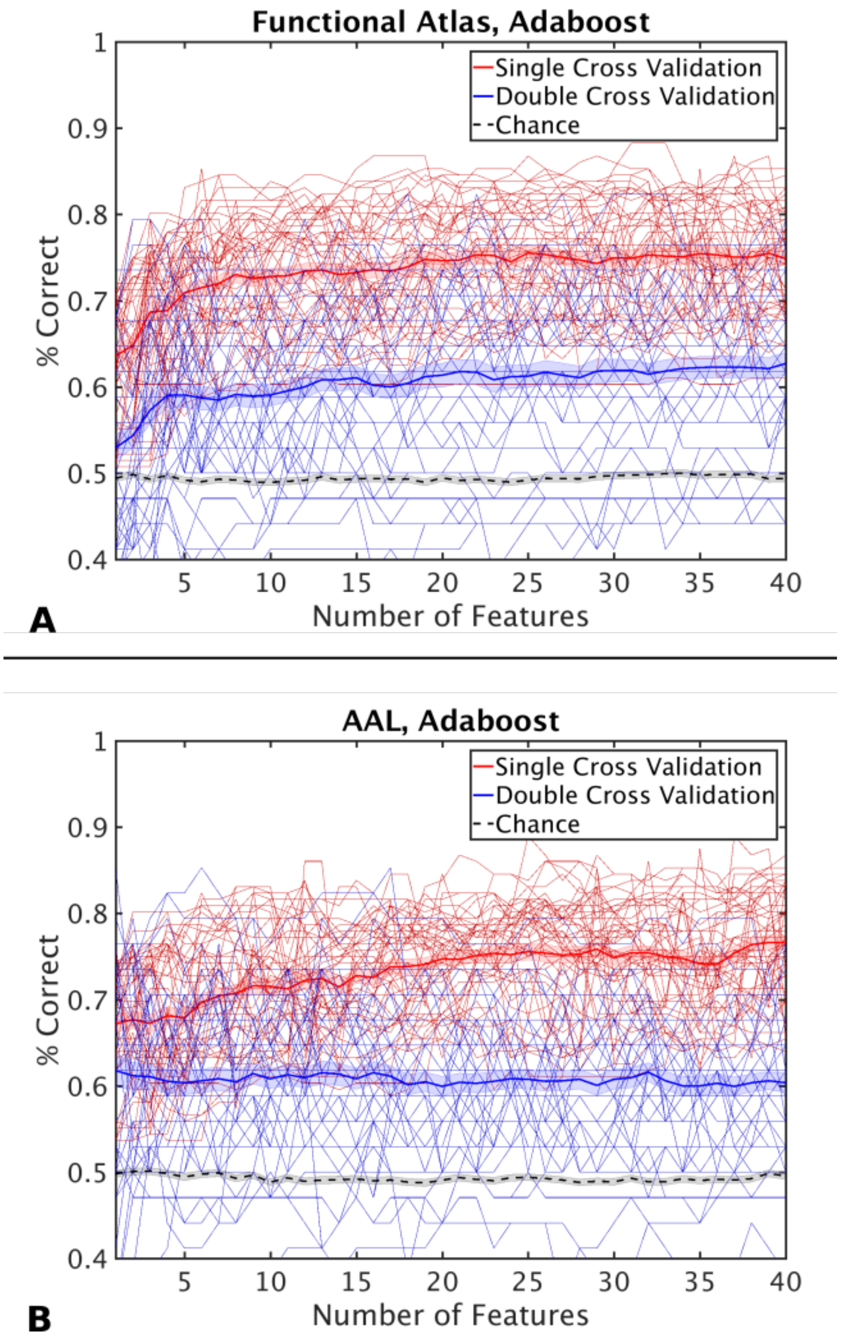

Supplementary Figure 2. Performance of Different Classifiers
Performance as a function of number of features for the adaptive boost (Adaboost) classifiers.
A) Performance of the Adaboost classifier when functional atlas was used to construct the network and extract features.
B) Performance of the Adaboost classifier when the AAL atlas was used to construct the network and extract features. Dotted line marks chance level.

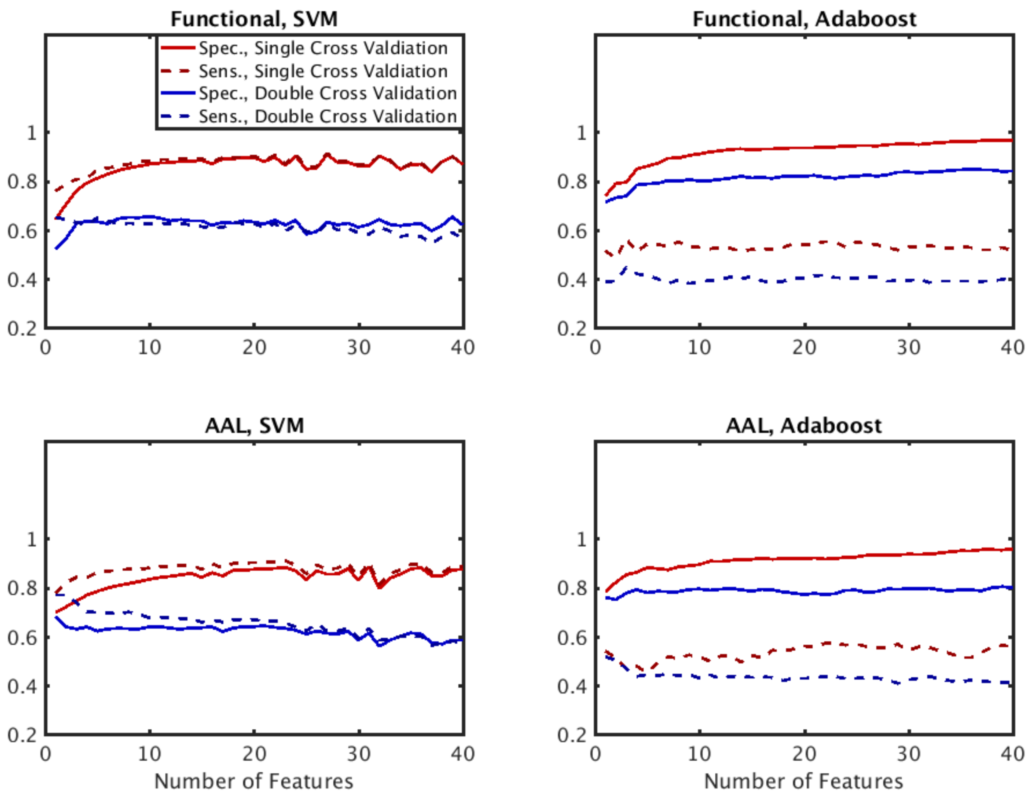

Supplementary Figure 3. Sensitivity and Specificity
Sensitivity and Specificity as a function of number of features for functional and AAL atlases and different classifiers.

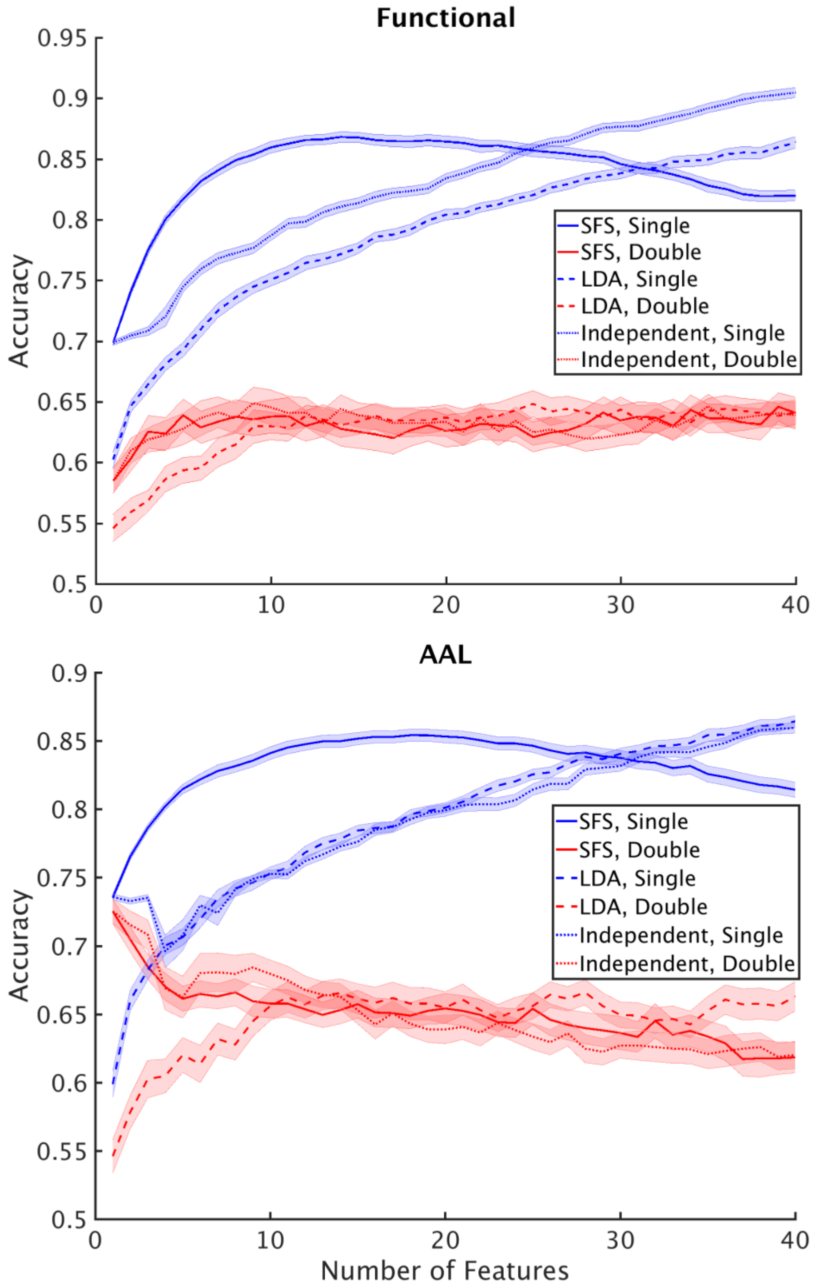

Supplementary figure 4. Feature Selection Method
Classification performance vs. number of features for different dimensional reduction methods for both functional (top) and AAL (bottom) atlases.

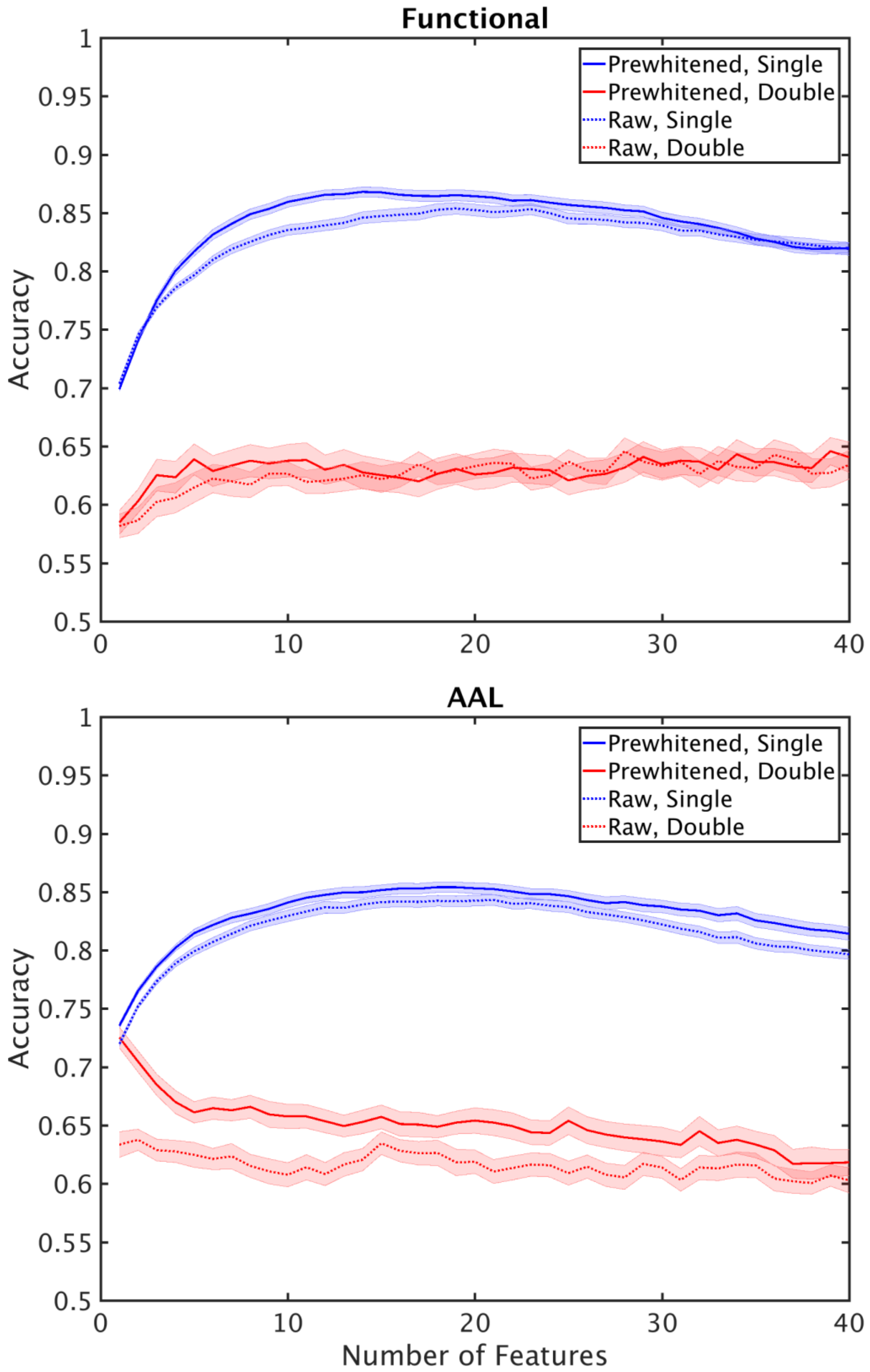

Supplementary figure 5. Raw vs Prewhitened
Classification accuracy vs. number of features when raw and prewhitened time series are used for construction of the network when the functional (top) and AAL (bottom) atlases were used for node definition.

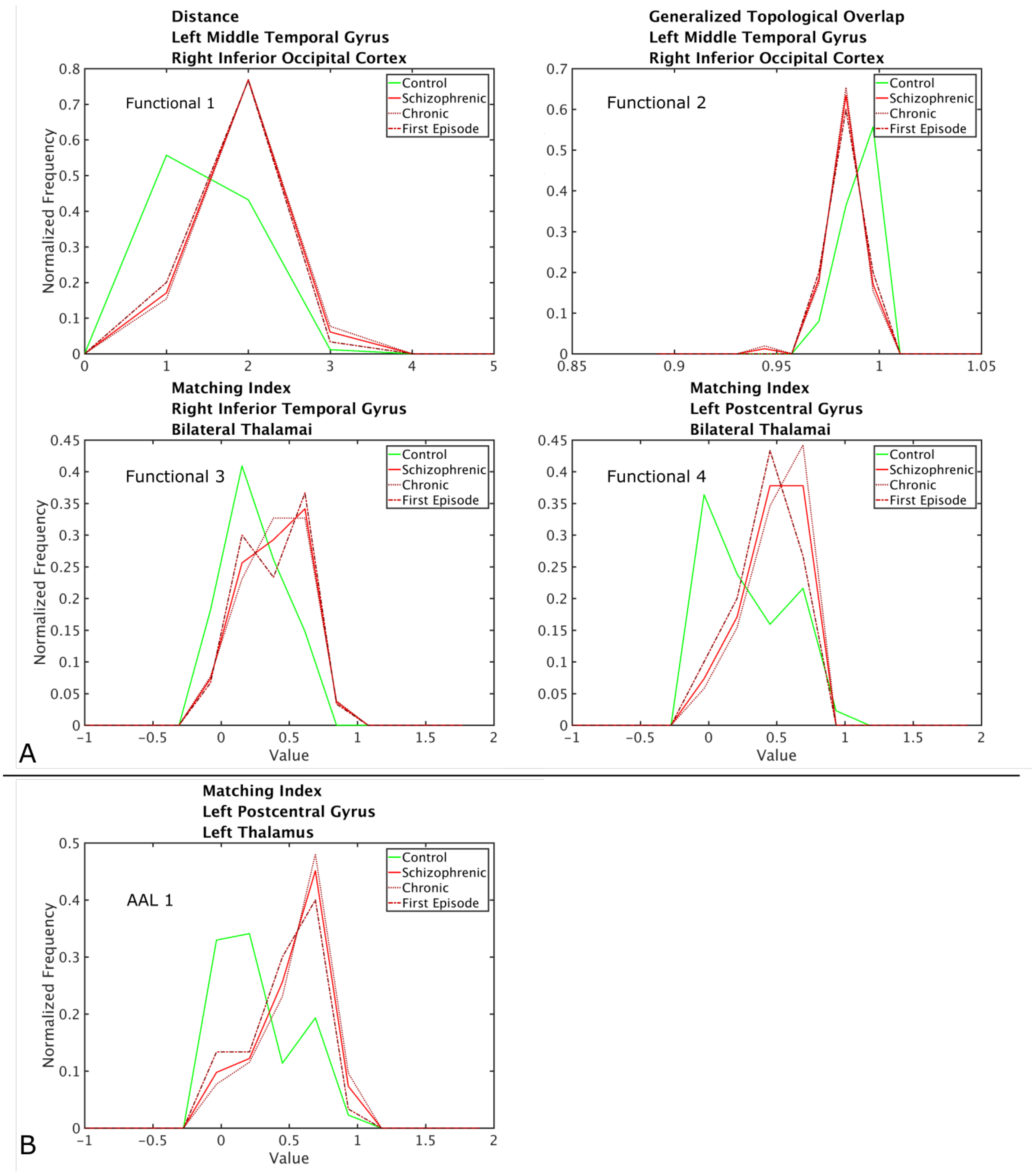

Supplementary Figure 6. Distribution of Top Features
Distribution of the top features across different subject groups for A) functional and B) AAL atlases.

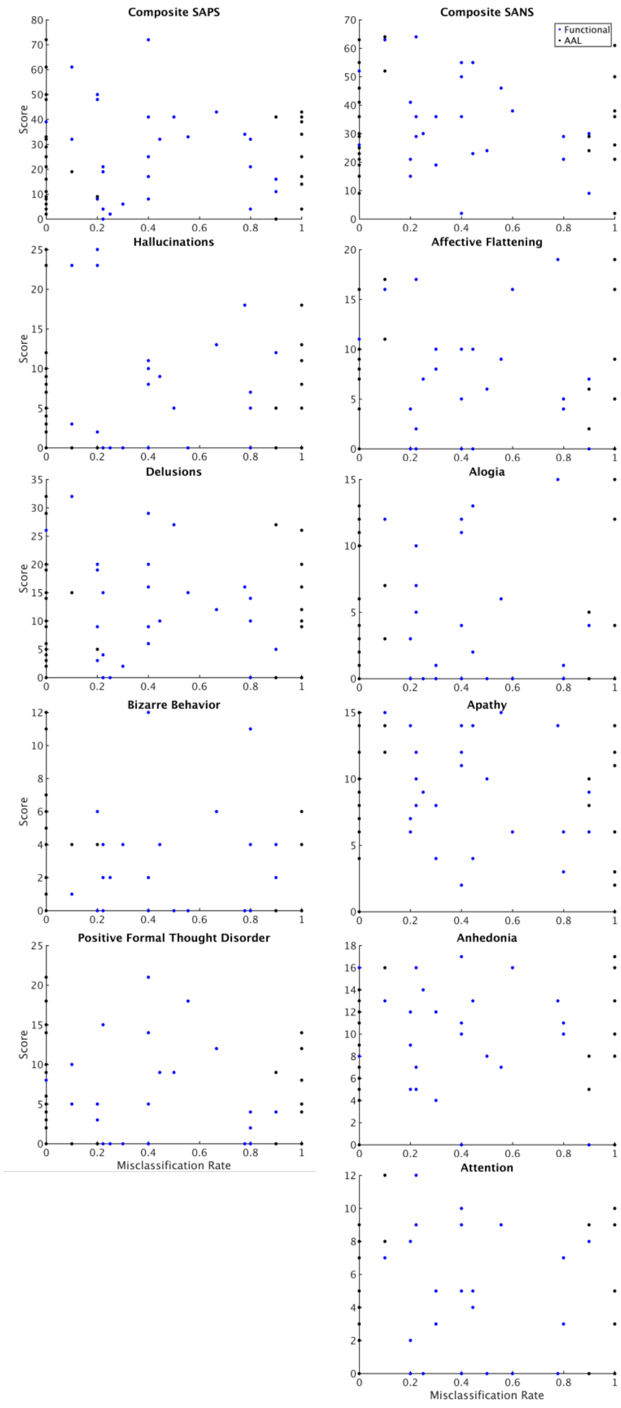

Supplementary Figure 7. Misclassification Rate vs. Severity
SANS and SAPS scores (y-axis) vs. Misclassification rate (x-axis) for the patient group for both atlases.
